# Ablation of kallikrein 7 (KLK7) in adipose tissue ameliorates metabolic consequences of high fat diet-induced obesity by counteracting adipose tissue inflammation in vivo

**DOI:** 10.1007/s00018-017-2658-y

**Published:** 2017-09-20

**Authors:** Konstanze Zieger, Juliane Weiner, Anne Kunath, Martin Gericke, Kerstin Krause, Matthias Kern, Michael Stumvoll, Nora Klöting, Matthias Blüher, John T. Heiker

**Affiliations:** 1 Institute of Biochemistry, Faculty of Biosciences, Pharmacy and Psychology, University of Leipzig, Brüderstr. 34, 04103 Leipzig, Germany; 2 Department of Medicine, University of Leipzig, Liebigstr. 20, 04103 Leipzig, Germany; 3 German Center for Diabetes Research (DZD), Munich, Germany; 4 Institute of Anatomy, University of Leipzig, Leipzig, Germany; 5 IFB Adiposity Diseases, University of Leipzig, Leipzig, Germany

**Keywords:** Adiposity, Insulin resistance, Metabolic syndrome, Serine proteases, Serpin

## Abstract

Vaspin is an adipokine which improves glucose metabolism and insulin sensitivity in obesity. Kallikrein 7 (KLK7) is the first known protease target inhibited by vaspin and a potential target for the treatment of metabolic disorders. Here, we tested the hypothesis that inhibition of KLK7 in adipose tissue may beneficially affect glucose metabolism and adipose tissue function. Therefore, we have inactivated the *Klk7* gene in adipose tissue using conditional gene-targeting strategies in mice. *Klk7*-deficient mice (AT*Klk7* ^−*/*−^) exhibited less weight gain, predominant expansion of subcutaneous adipose tissue and improved whole body insulin sensitivity under a high fat diet (HFD). AT*Klk7* ^−*/*−^ mice displayed higher energy expenditure and food intake, most likely due to altered adipokine secretion including lower circulating leptin. Pro-inflammatory cytokine expression was significantly reduced in combination with an increased percentage of alternatively activated (anti-inflammatory) M2 macrophages in epigonadal adipose tissue of AT*Klk7* ^−*/*−^. Taken together, by attenuating adipose tissue inflammation, altering adipokine secretion and epigonadal adipose tissue expansion, *Klk7* deficiency in adipose tissue partially ameliorates the adverse effects of HFD-induced obesity. In summary, we provide first evidence for a previously unrecognized role of KLK7 in adipose tissue with effects on whole body energy expenditure and insulin sensitivity.

**Electronic supplementary material:**

The online version of this article (doi:10.1007/s00018-017-2658-y) contains supplementary material, which is available to authorized users.

## Introduction

A primary risk factor associated with obesity is adipose tissue (AT) dysfunction which is characterized by extensive visceral and ectopic fat accumulation, adipocyte hypertrophy, a chronic low-grade AT inflammation with increased numbers of infiltrating AT macrophages (ATM) and initially local but ultimately systemic insulin resistance [1]. In consequence, there is a causal relationship between obesity and several major adverse health outcomes, such as type 2 diabetes, dyslipidemia, hypertension, cardiovascular and fatty liver disease as well as kidney diseases [2, 3]. Adipose tissue expresses and secretes a variety of adipokines, i.e., enzymes, cytokines, hormones, peptides and other biologically active molecules, which actively regulate whole body metabolism, energy homeostasis and inflammatory processes [4]. In consequence, AT dysfunction is reflected by an adipokine secretion pattern, promoting insulin resistance and a pro-inflammatory state.

The adipokine visceral AT serine protease inhibitor (vaspin) has been shown to improve glucose metabolism and insulin sensitivity and reduces food intake upon administration in pharmacological doses in different mouse models [5–9]. Genetically or high fat diet-induced insulin resistance is accompanied by increased expression of vaspin [7, 10]. Transgenic overexpression of vaspin in AT in mice markedly ameliorated glucose and insulin tolerance as well as adipose tissue inflammation under high fat diet-induced obesity [11]. Importantly, non-inhibitory vaspin mutants failed to improve glucose tolerance in insulin-resistant mice [9].

The serine protease kallikrein-related peptidase 7 (KLK7) is the only known target protease of vaspin so far and may therefore mediate vaspin´s effects in AT [9]. KLK7 (uniprot accession number P49862) is a member of the kallikrein subfamily of 15 closely related (chymo)trypsin-like serine proteases located on chromosome locus 19q13.4 in humans [12]. In rodents, the kallikrein gene family consists of more than 20 genes, including pseudogenes, but they share many similarities with respect to tissue-specific expression, regulatory mechanisms and function that demonstrate the evolutionary conservation across these species (reviewed in [13]). KLKs are known to participate in pathways regulating skin desquamation, kidney function, seminal liquefaction, synaptic neural plasticity and brain function (reviewed in [14]). KLK activities are tightly regulated and multiple mechanisms, such as pro-enzyme activation cascades, peptide as well as protein inhibitors (e.g., serpins and LEKTI), pH and metal ions fine-tune tissue-specific KLK activity (reviewed in [15]). As KLK proteases exert various important regulatory functions, they are promising targets in potential therapies of several diseases including respiratory diseases, neurodegeneration, skin-barrier dysfunction, inflammation and cancer (reviewed in [16]).

KLK7 was first identified as a protease involved in the desquamation process in the outermost layer of the skin [17, 18] and transgenic mice overexpressing human KLK7 display hyperkeratosis, epidermal thickness and cumulative appearances of immune cells in the dermis [19, 20]. Thus, its function in skin desquamation and role in the pathogenesis of inflammatory skin diseases such as psoriasis [21] and acne rosacea [22] are fairly well understood.

But, although it has been suggested that Klk7 may mediate beneficial effects of recombinant vaspin in mouse models of obesity and diabetes, the role of KLK7 in adipose tissue has not been systematically studied in vivo yet. Here, we tested the hypothesis that ablation of KLK7 in adipose tissue may beneficially affect glucose metabolism and adipose tissue function.

## Research design and methods

### Animal studies

All experiments conformed to the animal ethical law of the state Saxony, Germany, and were approved by the local animal ethics review board (Landesdirektion Sachsen, Leipzig, TVV23/12, T03/12, T08/15, Germany). Male mice were housed in a pathogen-free facility with a daylight cycle from 6.00 to 18.00 hours and 22 ± 2 °C, in groups of three to five animals. Animals were given a standard chow diet (chow; EV153, 3.3% from fat) or a Western diet (HFD) containing 55% calories from fat (E15772-34, Ssniff^®^, Germany). Mice permanently had free access to water and food; food restriction was only performed if required for an experiment. Female mice were investigated in parallel and showed a similar phenotype for key parameters of this study (Supplementary Fig. 1).

### Generation of adipocyte-specific *Klk7* knockout (KO) mice (AT*Klk7*^−/−^)

**Fig. 1:**
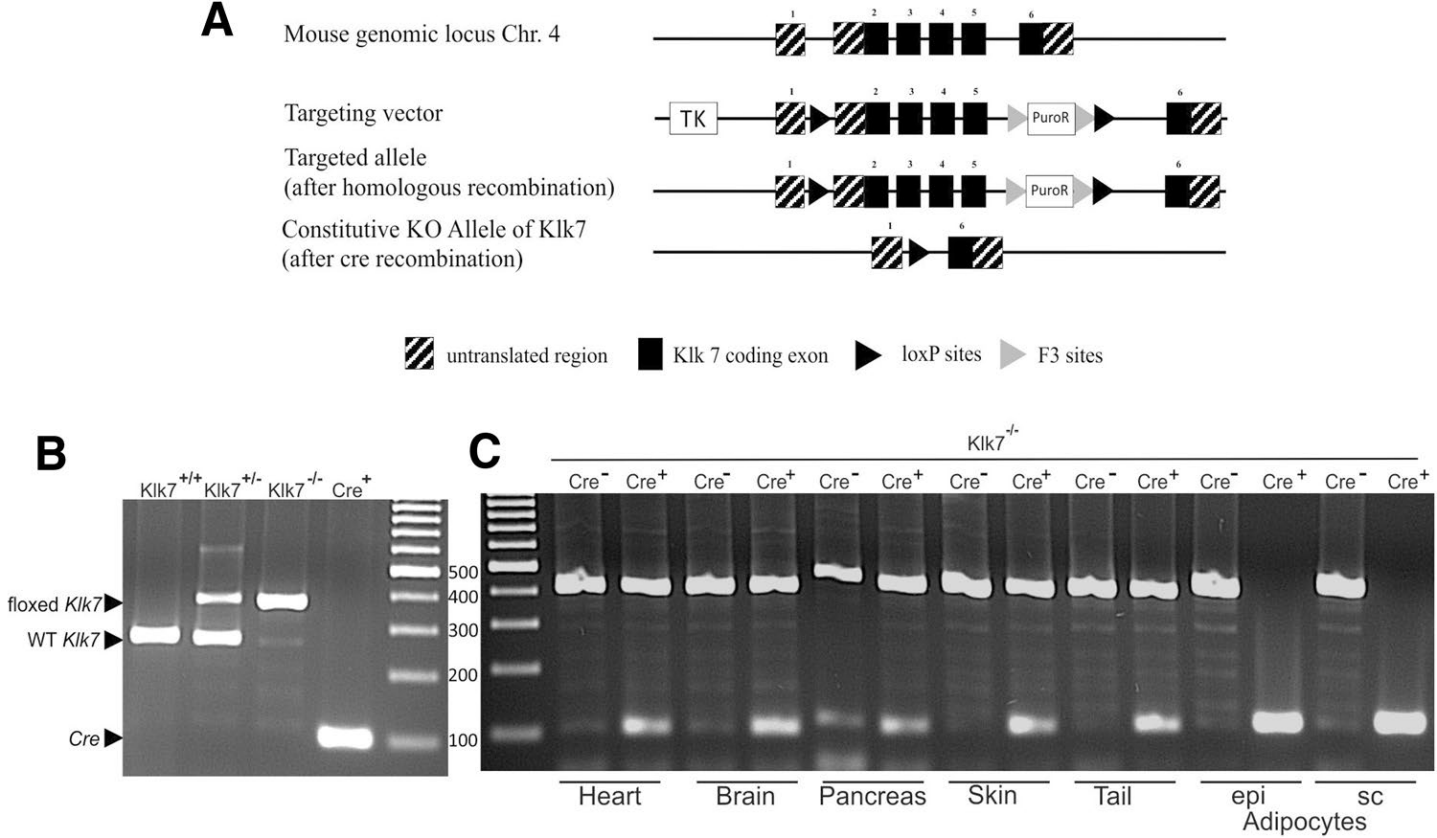
Targeting strategy for the generation of AT*Klk7* ^−*/*−^ mice. **a** Schematic representation of the loxP-flanked Klk7 allele before and after crossing with transgenic animals expressing Cre-recombinase under the Fabp4 promoter. The targeting vector consisted of 2 kb of the murine *Klk7* gene, comprising exon 2–5 flanked by loxP sites and two selection markers herpes simplex virus type 1 thymidine kinase (TK) and puromycin *N*-acetyltransferase (PuroP). F3 sites flanked the PuroP selection marker. The knockout (KO) allele, shown under the floxed allele, is characterized by the deletion of exons 2–5. The constitutive KO occurs by Cre-mediated deletion of exon 2–5 and the selection marker. **b** Genomic DNA from WT (Klk7^+/+^, predicted PCR product size of 276 bp), heterozygous floxed (Klk7^+/−^, predicted PCR products of 276 and 402 bp) or homozygous floxed Klk7 mice (Klk7^−/−^, predicted PCR product of 402 bp) was used as template for PCR of the floxed Klk7 allele and from Fabp4-Cre^+^ mice (Cre^+^, predicted PCR product of 100 bp) as template for PCR of the Cre allele. **c** Fabp4-Cre^+^_Klk7^−/−^ (ATKlk7^−/−^) mice exhibit an adipocyte/AT specific loss of the floxed *Klk7* allele. Genomic DNA from selected tissues (heart, brain, pancreas, skin, tail) and of isolated adipocytes from epigonadal (epi) and subcutaneous (sc) AT of Klk7^−/−^ mice with and without the Fabp4_Cre transgene was used as template for PCR of the Klk7^−/−^ and the Cre allele. Arrows indicate PCR products

The inactivation of the *Klk7* gene in adipose tissue (KO, AT*Klk7* ^−/−^) was obtained via the Cre-lox system for conditional gene-targeting strategies using animals with conditional (floxed) Klk7 (*Klk7* ^−*/*−^ by Taconic, Cologne, Germany) and adipose tissue-specific expression of Cre-recombinase under the fatty acid-binding protein 4 (Fabp4) promoter (*Fabp4*-*Cre* ^+^ *_Klk7* ^−*/*−^). In AT, Cre-recombinase mediates the deletion of all floxed alleles (Fig. 1a). ATKlk7 mice were maintained on a C57BL/6/NTac background. Littermates without conditional Klk7 (WT or *Fabp4*-*Cre* ^+^ *_Klk7* ^+*/*+^) or without Cre-recombinase (*Fabp4*-*Cre* ^−^ *_Klk7* ^−*/*−^) were used as controls (termed AT*Klk7* ^+/+^ throughout the manuscript).

### Genotyping of AT*Klk7*^−/−^ mice

Genotyping was accomplished by isolating genomic DNA from tail tips, using the INVISORB spin tissue mini kit (Stratec, Berlin, Germany) and quantified afterward by PCR. The following primers were used: Klk7 loxP sites, 5′-GGGATGTAGGATTATGAGTGAGC-3′ (forward) and 5′-CAGTCCAGTGAACTGCTCACC-3′ (reverse), as well as Cre-recombinase, 5′-GCGGTCTGGCAGTAAAAACTATC-3′ (forward) and 5′-GTGAAACAGCATTGCTGTCACTT-3′ (reverse). PCR was performed for 35 cycles, 95 °C for denaturation (loxP sites and Cre), 60 °C (loxP sites) or 56 °C (cre) for annealing and 72 °C (loxP sites and Cre) for elongation performed with the Fermentas Dream Taq Polymerase (Fermentas, St. Leon-Rot, Germany) and a Peltier Thermal Cycler PTC-200 (Bio-Rad, Hercules, CA, USA). With DNA from control mice, a 276 bp band and with DNA from AT*Klk7* ^−/−^ mice a 402 bp band were obtained on agarose gel.

### Phenotypic characterization

Ten to twelve male mice of each genotype [AT*Klk7* ^−/−^ and control littermates (AT*Klk7* ^+/+^)] were studied from an age of 7 up to 32 weeks under chow diet and from an age of 7 up to 20 weeks under HFD conditions. Body weight was recorded weekly and whole body fat mass as well as lean body mass were recorded in awake animals using an EchoMRI system at the beginning, middle and end of the studies (Echo Medical Systems, Houston, TX, USA). Intraperitoneal glucose (GTT) and insulin (ITT) tolerance test as well as hyperinsulinemic–euglycemic clamp studies were performed as previously described [9, 23]. In the chow group, ITT and GTT were analyzed at an age of 12 and 24 weeks and for HFD at an age of 12 and 14 weeks. For hyperinsulinemic–euglycemic clamp studies, catheters were implanted in the left jugular vein and clamps of five to eight males of each genotype (chow diet) were performed at the age of 20–24 weeks. Whole body energy metabolism was analyzed with an indirect calorimetry metabolic chamber system (TSE-Systems, Bad Homburg, Germany) at an age of 31 weeks (chow) or 21 weeks (HFD) as previously described [23].

Mice were killed at the age of 32 weeks (chow) or 20 weeks (HFD) by an overdose of anesthetic (Isofluran, Baxter, Unterschleißheim, Germany). Liver, heart, brain, pancreas, muscle, kidney, spleen, and epigonadal (epi) and subcutaneous (sc) AT were immediately removed. AT and liver were weighed and relative organ weights calculated in relation to body weights. Fasting blood glucose levels were obtained from whole venous blood samples using an automated glucose monitor (FreeStyle mini, Abbott GmbH, Ludwigshafen, Germany).

### Serum parameter analysis

Serum insulin (Mouse Insulin ELISA, Mercodia, Uppsala, Sweden), leptin (Mouse Leptin ELISA, Crystal Chem, Downers Grove, USA), C-peptide (Mouse C-peptide ELISA, ALPCO, Salem, USA), chemerin (Mouse Chemerin Quantikine ELISA, R&D Systems, Minneapolis, USA), adiponectin (Mouse Adiponectin ELISA, AdipoGen, San Diego, USA) and MCP-1 (Mouse/rat CCL2/JE/MCP-1 Quantikine ELISA, R&D Systems, Minneapolis, USA) levels were detected by ELISA according to the manufacturer’s protocol. For the analysis of free fatty acids (FFA), total cholesterol and triglycerides serum concentrations in fasting mice, an automatic chemical analyzer was used, provided by the Institute of Laboratory Medicine and Clinical Chemistry, Medical Department, University of Leipzig.

### RNA isolation and quantitative real-time PCR

RNA isolation and quantitative real-time PCR were performed as previously described [23]. RNA of primary adipocytes was isolated using InviTrap Spin Tissue RNA Mini Kit (Stratec Biomedical, Birkenfeld, Germany) as specified by the manufacturer. mRNA expression of genes listed in Supplementary Table 1 was determined and quantification of specific mRNA expression was calculated relative to *36B4* using the ΔΔCT method.

### Adipocyte isolation, adipocyte distribution, histology of AT and flow cytometry of AT macrophages

After sacrificing the animals, sc and epi fat depots were removed. Adipocytes were isolated via collagenase digestion (1 mg/ml) in a shaking water bath at 37 °C for 45 min. 200 µl aliquots of adipocytes were fixed with osmic acid at 37 °C for 48 h in the dark and subsequently counted in a Coulter counter (Multisizer III; Beckman Coulter Counter, Krefeld, Germany). Histology of AT as well as AT macrophage characterization was performed as described previously [24].

### Primary cell cultures

For primary adipocyte cultures, SVF from epigonadal, subcutaneous and intrascapular brown fat depots of 8- 12-week-old male mice (ATKlk7^−/−^ and ATKlk7^+/+^) were prepared, isolated and differentiated for 8 days as previously described [25]. Briefly, pooled tissue pieces were minced and digested in HEPES isolation buffer [0.1 M HEPES, 123 mM NaCl, 5 mM KCl, 1.3 mM CaCl_2_, 5 mM glucose, 4% BSA, 1% penicillin/streptomycin and 0.2% (w/v) collagenase II, pH 7.2] for 30 min at 37 °C. The cell suspension was filtered through a 100 µm nylon filter and subsequently incubated on ice for 15 min to let the mature adipocytes float up. SVF and mature adipocytes were separated and the SVF fraction was fine filtered through a 40 µm nylon filter followed by centrifugation (700*g*, 10 min, 4 °C). The medium was removed and preadipocytes were resuspended in erythrocyte lysis buffer for 5 min. After centrifugation, cells were suspended in ~0.5 ml culture medium (DMEM containing 10% FCS, 1% penicillin/streptomycin and 25 µg/ml sodium ascorbate). 0.4 ml cell suspension was seeded per 10 cm^2^ and grown at 37 °C and 5% CO_2_. The medium was changed on the first day and then every second day. After the cells reached confluency (day 0), the medium was changed to the differentiation cocktail (culture medium supplemented with 3 nM insulin, 1 nM T3, 1 µM rosiglitazone and 0.4 µg/ml dexamethasone) and experiments were carried out at indicated time points. Analysis of lipid accumulation using AdipoRed and fluorescence microscopy was performed as previously described [26].

### Statistical analysis

Data are given as mean ± SEM. All datasets were analyzed using a two-tailed unpaired Student’s *t* test or two-way ANOVA for repeated measurements and Sidak post-test to correct for multiple testing (GraphPad Prism software) and *P* values < 0.05 were considered as statistically significant. Sample sizes for every figure or table are presented in Supplementary Table 2.

## Results

### Generation of mice with adipose tissue-specific loss of kallikrein 7 (AT*Klk7*^−/−^)

To generate adipose tissue-specific knockout mice, mice homozygous for loxP-flanked *Klk7* allele (*Klk7* ^*flox/flox*^) were crossed with mice expressing Cre-recombinase under control of the adipose-specific fatty acid-binding protein 4 (Fabp4) promoter. The targeting strategy is shown in Fig. 1a. Mice were genotyped by PCR analysis of genomic DNA. Genomic DNA from WT (Klk7^+/+^, predicted PCR product size of 276 bp), heterozygous floxed (Klk7^+/−^, predicted PCR products of 276 and 402 bp) or homozygous floxed Klk7 mice (Klk7^−/−^, predicted PCR product of 402 bp) were used for PCR of the floxed Klk7 allele and showed the predicted bands for each genotype (Fig. 1b). Genomic DNA from Fabp4-Cre^+^ mice used for PCR of the Cre allele also yielded the predicted PCR product of 100 bp (Fig. 1b). Knockdown and AT specificity of the Fabp4-Cre^+^_Klk7^−/−^ (AT*Klk7* ^−*/*−^) was examined by PCR analysis in various tissues (heart, brain, pancreas, skin, tail, isolated adipocytes from epi and sc AT) for AT*Klk7* ^−/−^ and control mice (Fig. 1c). AT*Klk7* ^−*/*−^ mice exhibit an adipocyte/AT specific loss of the floxed Klk7 allele (Fig. 1c).

### AT Klk7 deficiency prevents extensive epigonadal AT accumulation upon HFD

**Fig. 2:**
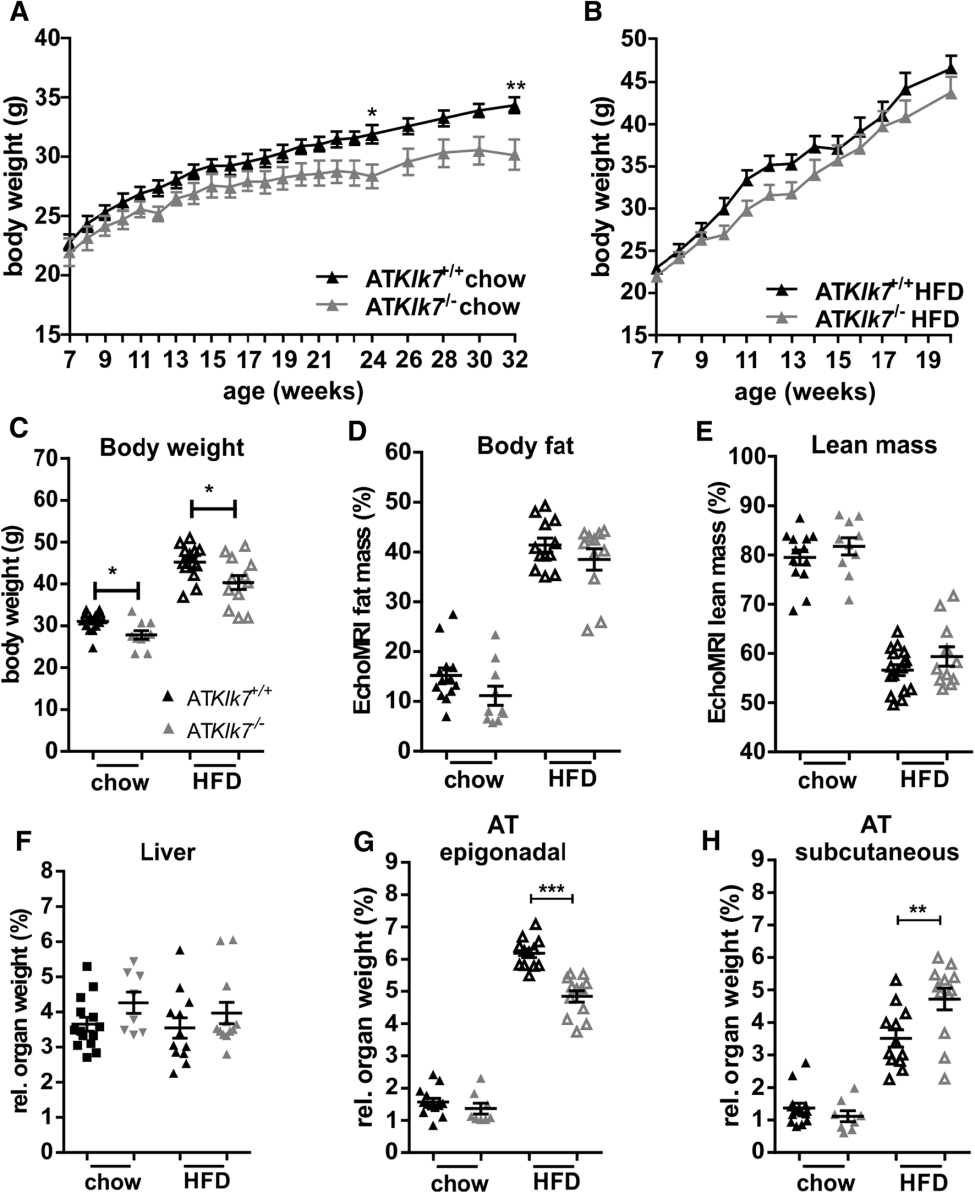
Klk7 deficiency in adipose tissue causes a redistribution of adipose tissue from epigonadal to subcutaneous AT depots under high fat diet conditions independently of total body fat mass gain. **a**, **b** Body weight gain and **c** final body weight of AT*Klk7* ^−/−^ and control littermates fed a regular chow diet for 26 weeks (**a**) or a 55% high fat diet (HFD) for 14 weeks (**b**), starting at the age of 6 weeks (*n* = 9–16). **d**, **e** The percentage of fat mass and lean body mass as determined by EchoMRI at the end of the studies under chow (at age 26 weeks) and HFD conditions (20 weeks of age; *n* = 10–12). **f**–**h** Relative organ weights of liver, epigonadal and subcutaneous white adipose tissue depots (*n* = 8–14) at the end of chow and HFD studies. AT*Klk7* ^−/−^ exhibits altered fat distribution with increased subcutaneous and decreased epigonadal AT mass. Data are represented as mean ± SEM and for each diet condition differences between genotypes were tested for statistical significance by a two-tailed Student’s *t* test; *P < 0.05, **P < 0.01, ***P < 0.001

Comparison of AT*Klk7* ^−/−^ and controls under normal chow and HFD demonstrated that Klk7-deficient mice gain less weight both under chow and HFD conditions (Fig. 2a, b) and exhibit ~10% less body weight at the end of the study (Fig. 2c). Body composition analyses using EchoMRI revealed that under both chow and HFD conditions, total body fat and lean mass were not significantly different between AT*Klk7* ^−/−^ and control mice (Fig. 2d, e). Noteworthy, relative liver weights were not different between the genotypes under both conditions (Fig. 2f). Importantly, upon HFD, AT*Klk7* ^−/−^ mice accumulate significantly more sc AT (4.7 vs 3.5%; Fig. 2g) and significantly less epigonadal fat compared to controls (4.8 vs 6.2%; Fig. 2h). AT Klk7 deficiency did not cause alterations in circulating parameters of glucose and lipid metabolism or insulin sensitivity (Table 1). However, after 14 weeks of HFD, AT*Klk7* ^−/−^ mice exhibit significantly lower fasted serum concentrations of leptin, insulin, and in tendency C-peptide (Table 1). Together, these observations indicate that KLK7 deficiency promotes a shift in adipose tissue distribution with enhanced sc AT expandability and prevention of extensive epi AT accumulation under HFD in AT*Klk7* ^−/−^ mice.

**Table 1:** Fasting serum parameters in AT*Klk7* ^+/+^ and AT*Klk7* ^−/−^ under standard chow or HFD conditions

Trait	Chow		HFD	
	AT*Klk7* ^+/+^	AT*Klk7* ^−/−^	AT*Klk7* ^+/+^	AT*Klk7* ^−/−^
Glucose (mmol/l)	3.77 ± 0.25	4.58 ± 0.48	6.29 ± 0.28	6.75 ± 0.42
HbA1c (%)	4.26 ± 0.08	4.29 ± 0.10	4.55 ± 0.08	4.70 ± 0.04
Insulin (ng/ml)	0.24 ± 0.05	0.25 ± 0.06	4.66 ± 0.51	**2.94** **±** **0.43**
C-peptide (ng/ml)	0.75 ± 0.13	1.09 ± 0.11	3.49 ± 0.31	2.73 ± 0.29
Leptin (µg/ml)	0.29 ± 0.04	0.32 ± 0.09	2.43 ± 0.09	**1.66** **±** **0.25**
Adiponectin (µg/ml)	62.28 ± 4.58	60.57 ± 3.65	48.20 ± 2.02	47.35 ± 2.08
Leptin/adiponectin ratio	0.04 ± 0.01	0.03 ± 0.01	1.37 ± 0.11	**0.85** **±** **0.17**
Chemerin (µg/ml)	n.d.	n.d.	191.33 ± 14.11	182.94 ± 9.09
FFA (mmol/l)	1.54 ± 0.12	1.33 ± 0.11	1.50 ± 0.10	1.41 ± 0.07
Total cholesterol (mmol/l)	2.33 ± 0.14	2.47 ± 0.06	4.35 ± 0.50	4.65 ± 0.24
Triglycerides (mmol/l)	1.29 ± 0.06	1.16 ± 0.07	1.61 ± 0.09	1.50 ± 0.03

Values are presented as mean ± SEM and tested for statistical significance by the two-tailed Student’s *t* test (AT*Klk7* ^+/+^ vs. AT*Klk7* ^+/+^ under HFD, *n* = 8–10 per group)

Significantly different values at *P* < 0.05 are in bold

### AT Klk7 deficiency increases energy expenditure and food intake under HFD

**Fig. 3:**
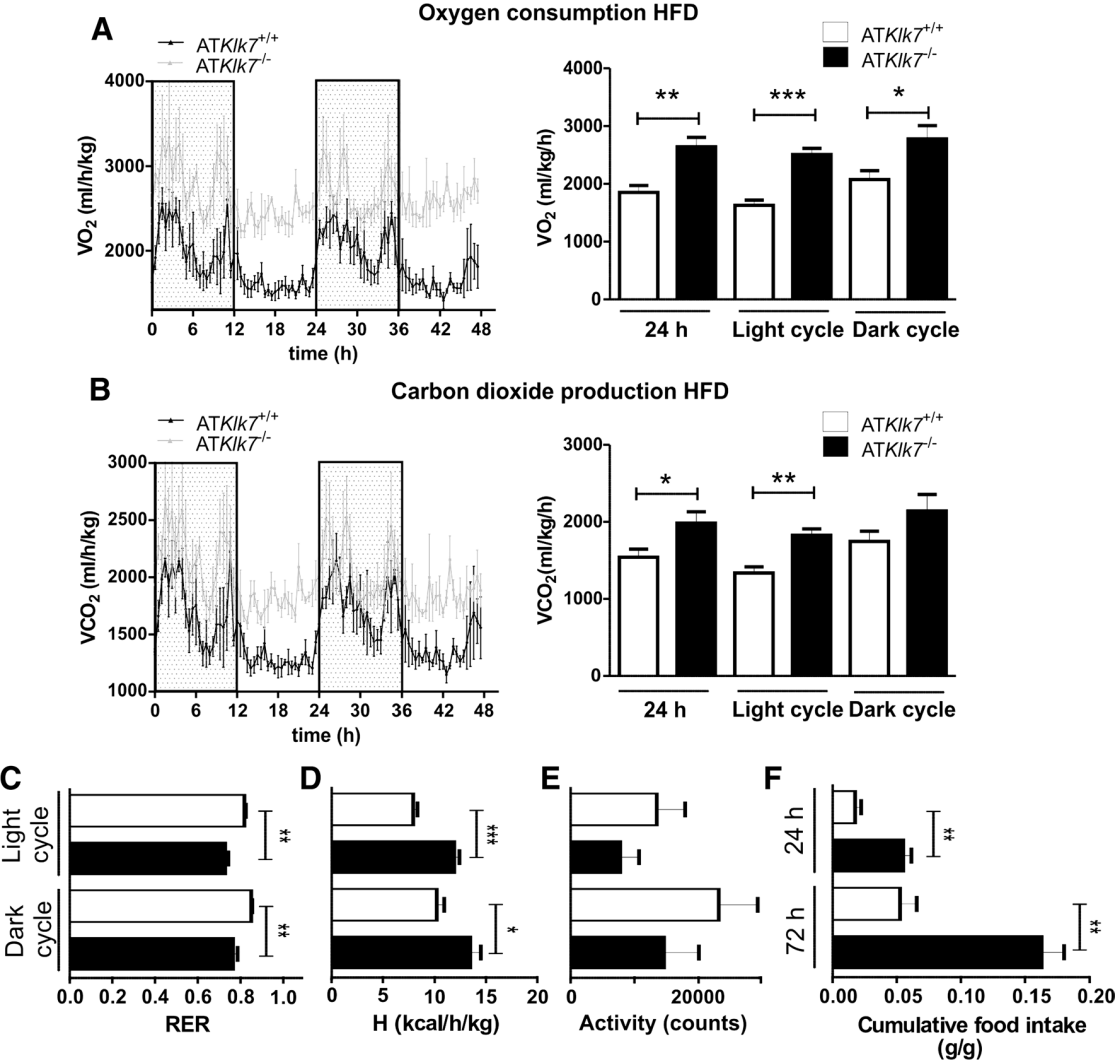
Higher energy expenditure and food intake in AT*Klk7* ^−/−^ compared to control mice under HFD. Oxygen consumption (**a**) and carbon dioxide production (**b**) over a period of 48 h were significantly higher in AT*Klk7* ^−/−^ compared to control (AT*Klk7* ^+/+^) mice (*n* = 4 per genotype). **c** Respiratory exchange rate (RER) was lower in AT*Klk7* ^−/−^ mice, indicating better adaptation to lipid utilization. **d** Net energy expenditure (**h**) was higher in AT*Klk7* ^−/−^ compared to controls. **e** Physical activity was not significantly different between AT*Klk7* ^−/−^ and control mice. **f** Cumulative food intake was significantly higher in AT*Klk7* ^−/−^ compared to control mice, both after 24 h and after 72 h. Data are presented as grams food per gram body weight. Data are represented as mean ± SEM (*n* = 4 per genotype) and for dark or light cycle differences between genotypes were tested for statistical significance by a two-tailed Student’s *t* test; *P < 0.05, **P < 0.01, ***P < 0.001

Since AT*Klk7* ^−/−^ mice exhibit lower body weights both under chow and HFD conditions, we next investigated food intake, activity and energy expenditure in metabolic chamber studies over 72 h. Indirect calorimetry revealed higher oxygen consumption (VO_2_) and carbon dioxide production (VCO_2_) in AT*Klk7* ^−/−^ compared to control mice after HFD (Fig. 3a, b). Both were significantly higher over the whole day 24 h span, in particular during the light phase. Furthermore, in AT*Klk7* ^−/−^ mice, the respiratory exchange ratio (RER) was significantly decreased both during the day and night (day: 0.73 vs 0.82, *P* = 0.0016; night: 0.76 vs. 0.85, *P* = 0.0072), indicating a shift from carbohydrate to fatty acid metabolism (Fig. 3c). Whereas AT*Klk7* ^−/−^ mice exhibited higher energy expenditure, locomotor activity was not different between both genotypes (Fig. 3d, e). Surprisingly, cumulative food intake was significantly higher in AT*Klk7* ^−/−^ compared to control mice (Fig. 3f). These findings provide direct evidence that Klk7 deficiency in adipose tissue results in higher energy expenditure, together with increased food intake and improved fatty acid oxidation. These paradoxical findings could be due to alterations in adipose tissue function, which are reflected by lower leptin serum concentrations in AT*Klk7* ^−/−^ compared to control mice after HFD (Table 1). However, adiponectin serum concentrations were not affected by AT Klk7 deficiency, suggesting that Klk7 in adipose tissue regulates adipokine release in a specific manner. AT*Klk7* ^−/−^ mice thus seem to better adapt to an HFD.

### Loss of *Klk7* in AT ameliorates diet-induced whole body insulin resistance

**Fig. 4:**
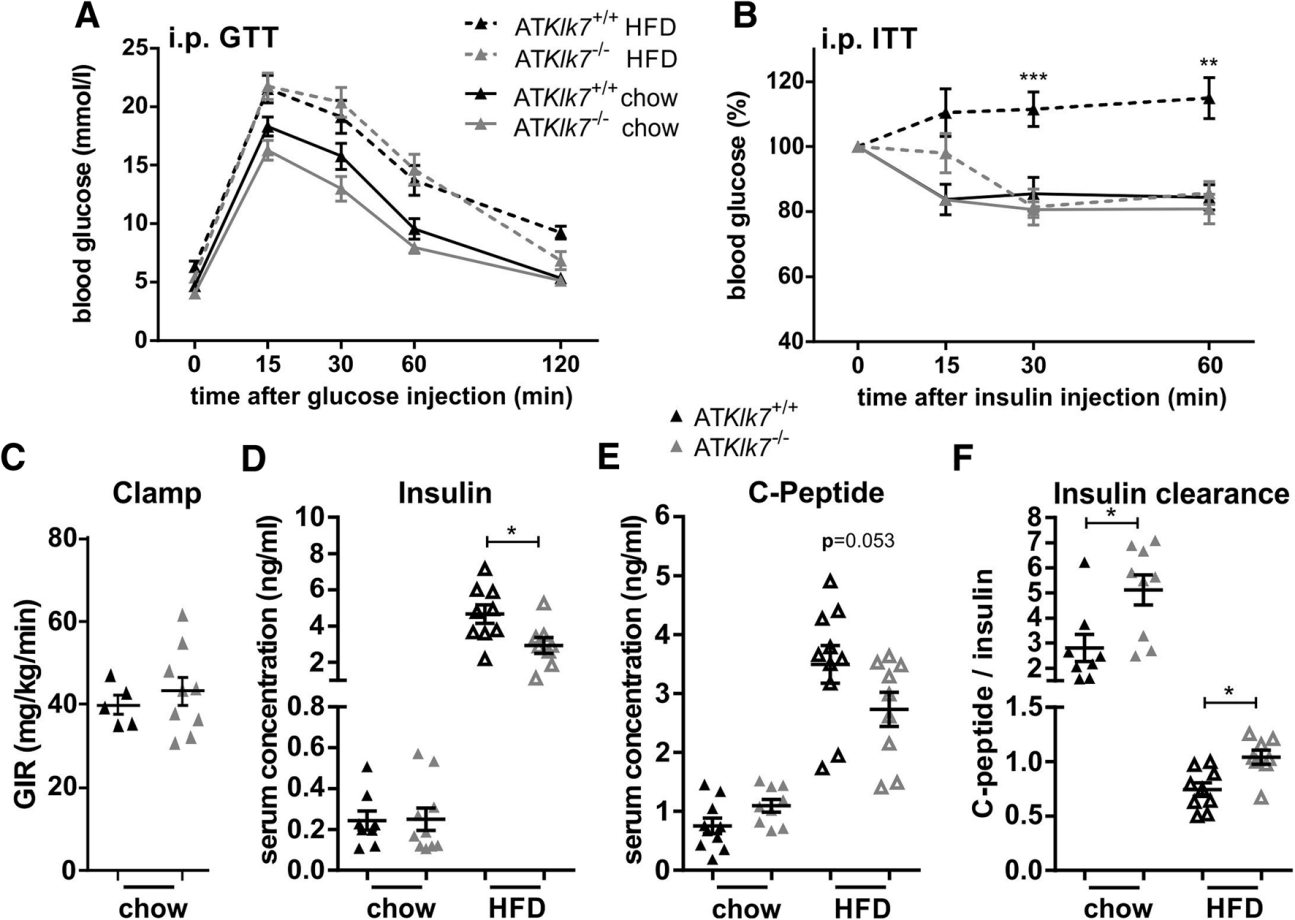
Parameters of glucose metabolism, insulin sensitivity and adipokine secretion in AT*Klk7* ^−/−^ mice. **a** Intraperitoneal glucose tolerance (GTT) and **b** insulin tolerance (ITT) after chow (24 weeks of age) and HFD (14 weeks of age). Glucose tolerance was not different between AT*Klk7* ^−/−^ and control mice, both under chow and HFD conditions. Under HFD, Klk7 deficiency in AT resulted in significantly improved insulin sensitivity (*n* = 10–13). **c** Hyperinsulinemic–euglycemic clamp shows a slight increase in glucose infusion rate for AT*Klk7* ^−/−^ compared to control mice under chow diet (*n* = 5–9). Mice were fasted overnight and insulin (**d**) and C-peptide (**e**) levels were measured in serum and the ratio was calculated as a surrogate parameter of insulin clearance (**f**) in AT*Klk7* ^−/−^ and control mice under chow (32 weeks of age) and HFD (20 weeks of age) conditions (*n* = 8–10). Data are presented as mean ± SEM and for each diet condition differences between genotypes were tested for statistical significance by a two-tailed Student’s *t* test; *P < 0.05, **P < 0.01, ***P < 0.001

Both control and AT*Klk7* ^−/−^ mice develop a mild fasting hyperglycemia in response to HFD (Table 1), but HbA1c was not different between the genotypes (Table 1). Under chow conditions, glucose tolerance and insulin tolerance were similar between AT*Klk7* ^−/−^ and control mice (Fig. 4a, b). Glucose tolerance was marginally improved and also hyperinsulinemic–euglycemic clamp studies indicated a slight, but not significant improvement of insulin sensitivity for AT*Klk7* ^−/−^ mice under a chow diet (Fig. 4c). Although under HFD-fed conditions glucose tolerance deteriorated in a parallel fashion in AT*Klk7* ^−/−^ and control mice (Fig. 4a), AT*Klk7* ^−/−^ mice remained insulin sensitive as indicated by insulin tolerance tests (Fig. 4b) and lower fasting insulin and C-peptide serum concentrations (Fig. 4c, d, Table 1). Significantly higher C-peptide/insulin ratios in AT*Klk7* ^−/−^ mice indicated improved insulin clearance (Fig. 4f). Also, the leptin/adiponectin ratio, a surrogate marker to assess the metabolic syndrome, was significantly lower in AT*Klk7* ^−/−^ mice under HFD (Table 1).

### Altered subcutaneous adipocyte morphology, subcutaneous AT expandability and expression of key AT genes

**Fig. 5:**
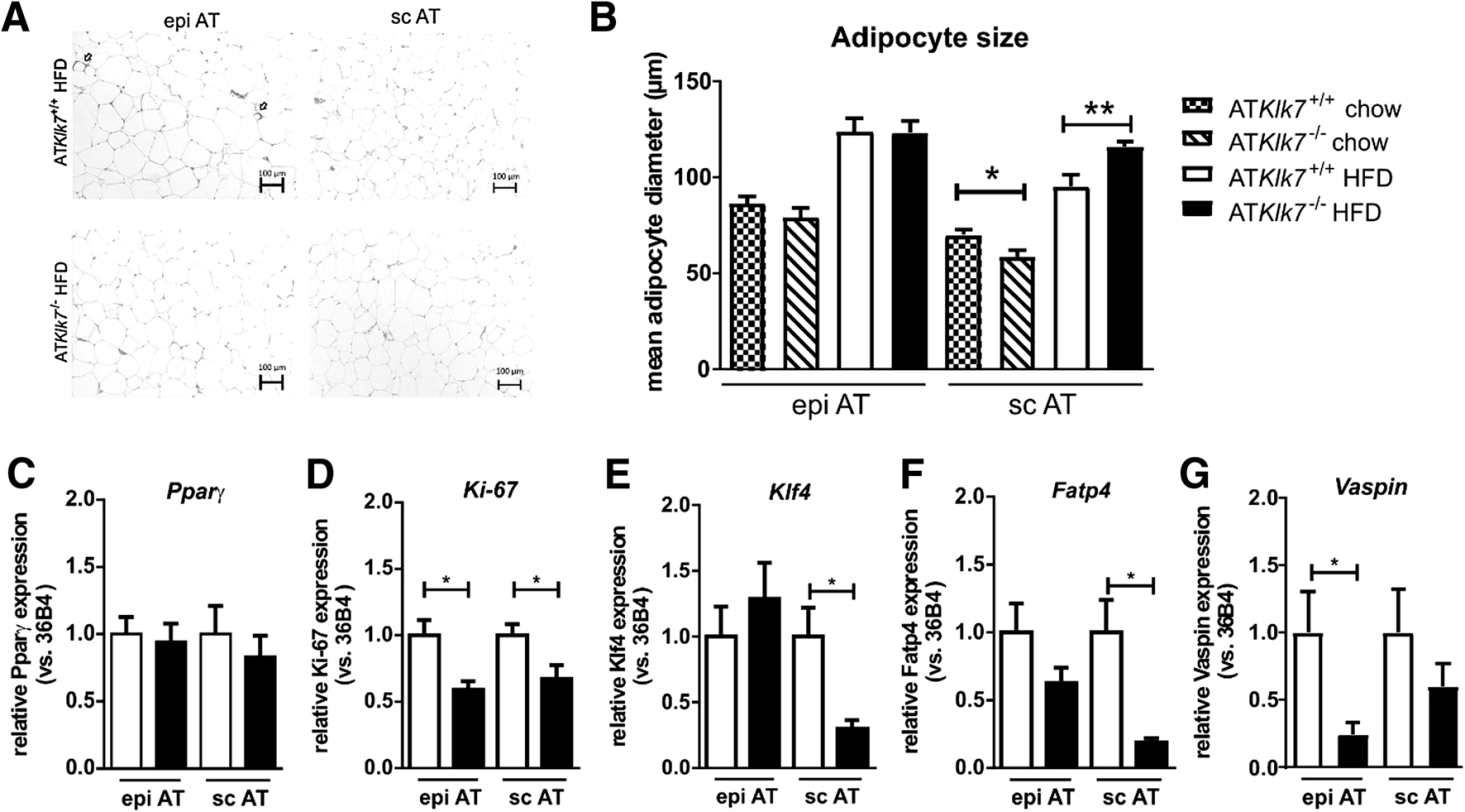
Adipose tissue morphology and adipocyte size were altered in AT*Klk7* ^−/−^ mice. **a** Histological analyses by H&E staining of epigonadal AT (left panel) and subcutaneous AT (right panel) suggest hypertrophy of subcutaneous adipocytes of AT*Klk7* ^−/−^ compared to control mice after HFD. **b** Mean epigonadal (epi) and subcutaneous (sc) adipocyte diameters were analyzed using a multisizer (Beckman Coulter) and confirmed HFD-induced hypertrophy of subcutaneous adipocytes. The mean size of epigonadal adipocytes was not different between the genotypes, both under chow and HFD conditions, whereas expandability of sc AT seems to be improved in AT*Klk7* ^−/−^ compared to control mice (*n* = 9–14). **c**–**g** Expression of *Pparγ*, *Ki*-*67*, *Klf4*, *Fatp4* and *vaspin* mRNA was examined by quantitative RT-PCR (*n* = 7–8). Data are represented as mean ± SEM and for each AT depot differences between genotypes were tested for statistical significance by a two-tailed Student’s *t* test; *P < 0.05, **P < 0.01

To investigate whether the altered AT distribution in AT*Klk7* ^−/−^ mice under HFD was accompanied by changes in adipocyte morphology, we analyzed AT histology and measured adipocyte size distribution. Hematoxylin/eosin (HE) staining of the subcutaneous and epigonadal AT depots clearly showed diet-induced hypertrophy of adipocytes independent of genotype and AT depot (Fig. 5a). The mean size of epigonadal adipocytes was not different between the genotypes both under chow and HFD conditions, whereas expandability of sc AT seems to be increased in AT*Klk7* ^−/−^ compared to control mice (Fig. 5b).

Marker gene expression analyses did not indicate differences in AT *Pparγ* expression (Fig. 5c), but revealed significantly lower expression of the proliferation marker *Ki*-*67* in AT depots upon HFD in AT*Klk7* ^−/−^ mice (Fig. 5d). Interestingly, we found the expression of Krüppel-like factor 4 (*Klf4*) and fatty acid transport protein 4 (*Fatp4*) to be significantly reduced in subcutaneous AT of AT*Klk7* ^−/−^ mice vs controls under HFD (Fig. 5e, f). Expression of the KLK7 inhibitor vaspin in AT was decreased, with a significant reduction in epi AT of HFD fed in AT*Klk7* ^−/−^ mice (Fig. 5g).

### AT Klk7 deficiency does not affect adipogenesis in primary adipocytes

**Fig. 6:**
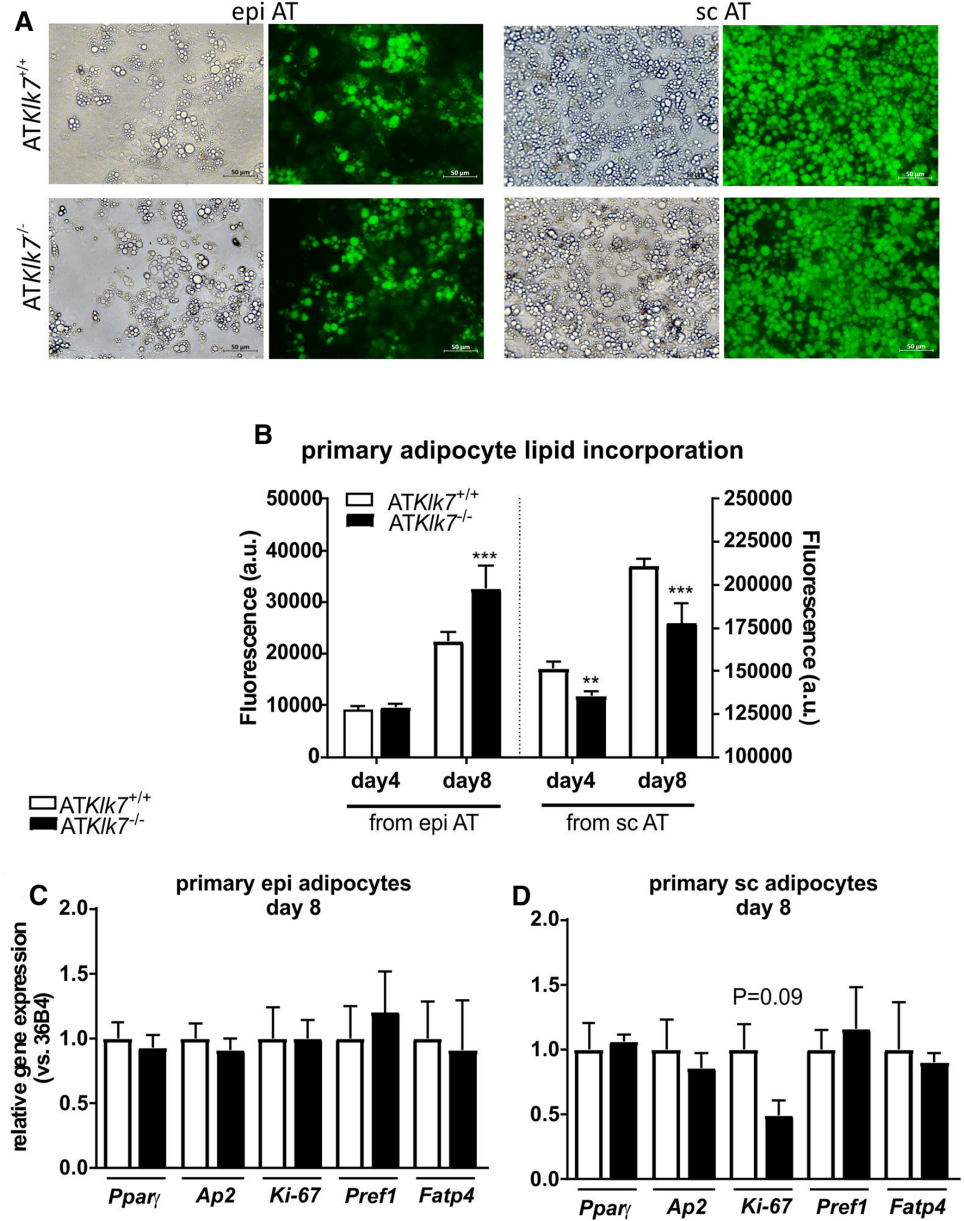
AT *Klk7* deficiency in primary adipocytes. **a** Fluorescence microscopy of fully differentiated primary adipocytes from epi and sc AT. Lipid droplets were stained with the lipophilic AdipoRed reagent. Pictures were taken at day 8 after induction of differentiation. **b** Quantification of lipid accumulation using the AdipoRed assay in primary adipocytes from epi and sc AT (*n* = 5 wells). mRNA expression of marker genes of adipogenesis at day 8 after induction of differentiation was examined in primary cells of epi (**c**) and sc (**d**) AT by quantitative RT-PCR (*n* = 3). Data are represented as mean ± SEM and differences between genotypes were tested for statistical significance by a two-tailed Student’s *t* test; **P < 0.01, ***P < 0.001

Primary adipocytes were differentiated from the stroma-vascular fraction (SVF) of epi AT, sc AT and BAT from both control and AT*Klk7* ^−/−^ mice fed a chow diet. All SVF fractions differentiated into mature adipocytes, and 8 days after induction of adipogenesis the vast majority of SVF cells from sc AT differentiated into adipocytes (Fig. 6a). Density of differentiated primary adipocytes from epi AT was less for both genotypes (Fig. 6a). Quantification of intracellular lipid accumulation revealed a higher lipid content in terminally differentiated primary adipocytes from epi AT of AT*Klk7* ^−*/*−^ mice compared to controls, while primary adipocytes from sc AT of AT*Klk7* ^−*/*−^ mice exhibited slightly but significantly reduced lipid content compared to controls (Fig. 6b, note the different *y*-axes). In line with the microscopy data, the fluorescence signal was ~tenfold lower for primary adipocytes from epi AT. Analysis of marker genes of adipogenesis did not show differences in primary epigonadal adipocytes (Fig. 6c). Thus, under chow conditions, KLK7 does not seem to significantly affect differentiation.

### AT Klk7 deficiency has minor effects on BAT and WAT browning

**Fig. 7:**
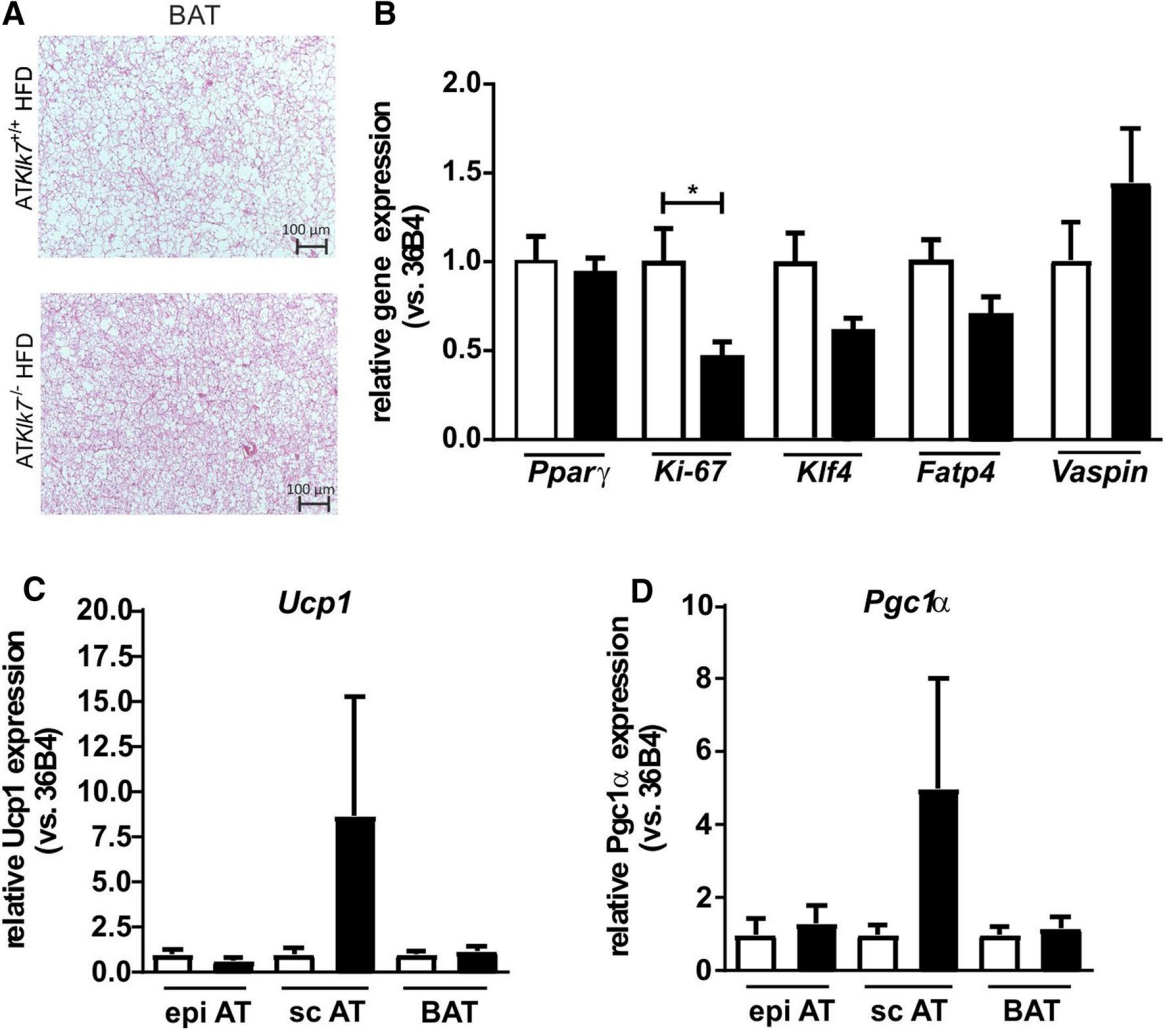
AT Klk7 deficiency, BAT morphology and AT browning. **a** H&E staining of BAT shows reduced hypertrophy and whitening of brown adipocytes in AT*Klk7* ^−/−^ compared to control mice after HFD. **b** Expression of *Pparγ*, *Ki*-*67*, *Klf4*, *Fatp4* and *vaspin* mRNA was examined by quantitative RT-PCR (*n* = 7–8). Expression of *Ucp1* (**c**) and *Pgc1α* (**d**) mRNA was examined by quantitative RT-PCR in epi, sc and brown AT (*n* = 7–8). Data are represented as mean ± SEM and differences between genotypes were tested for statistical significance by a two-tailed Student’s *t* test; *P < 0.05

We have further investigated adipocyte morphology and adipogenic gene expression in BAT under HFD in addition to the WAT depots. HE staining of BAT revealed a marked increase in BAT whitening in control mice compared to AT*Klk7* ^−*/*−^ mice with smaller lipid droplets and less adipocyte hypertrophy (Fig. 7a). As in the WATs, there were no differences in AT *Pparγ* expression, but a significantly lower expression of proliferation marker *Ki*-*67* in HFD-fed AT*Klk7* ^−*/*−^ mice (Fig. 7b). *Klf4* and *Fatp4* expression were lower, but the differences were not significant (Fig. 7b). In contrast to WAT depots, the expression of KLK7 inhibitor vaspin was slightly increased in AT*Klk7* ^−*/*−^ mice (Fig. 7b). Gene expression of key BAT markers *Ucp1* and *Pgc1α* expression was unchanged in BAT and epi AT, but increased in sc AT of AT*Klk7* ^−*/*−^ mice under HFD, though these increases were very inhomogeneous and not statistically significant (Fig. 7c).

### Adipose tissue inflammation and polarization of ATMs is altered in AT*Klk7*^−/−^ mice

**Fig. 8:**
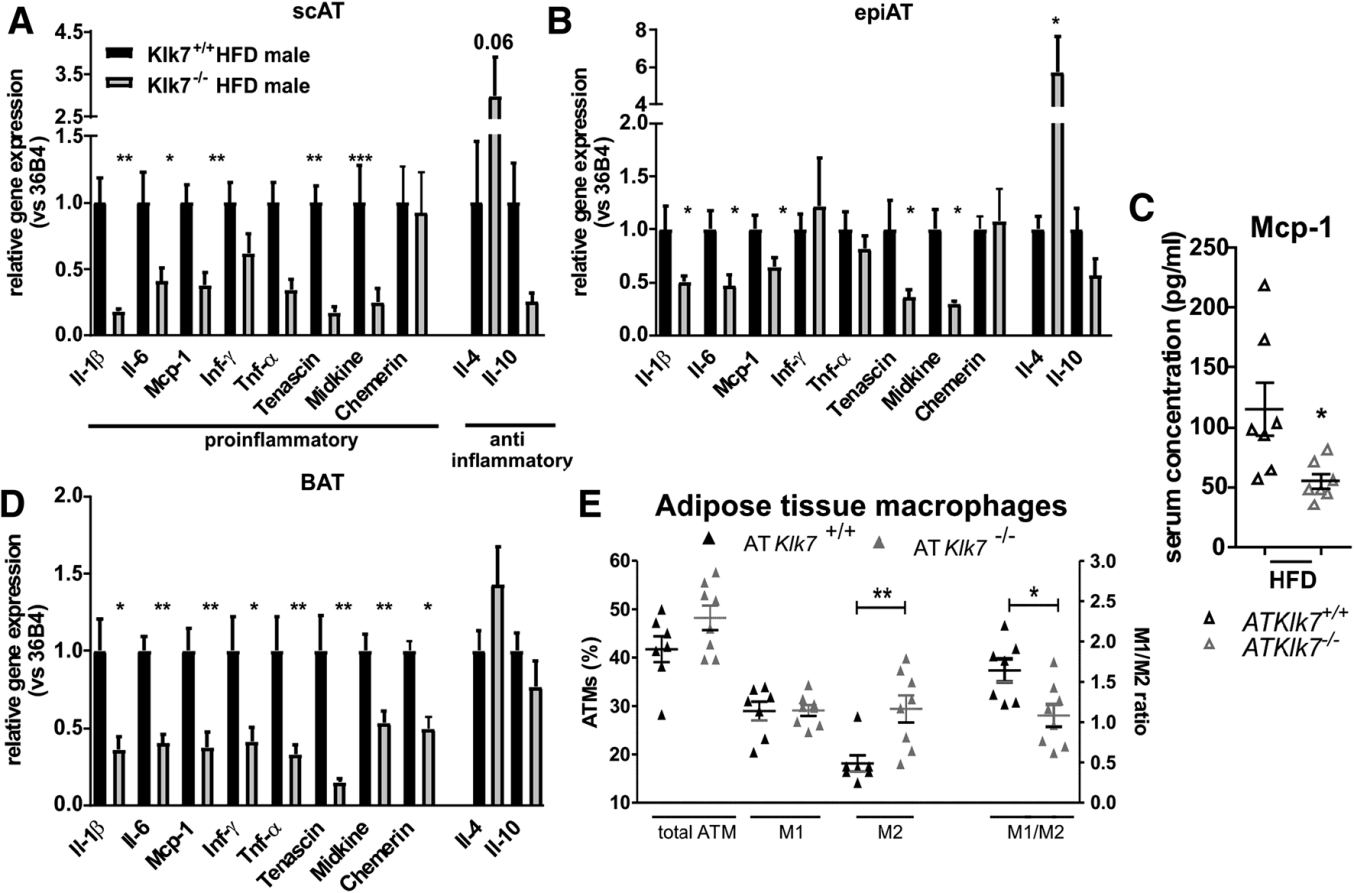
AT*Klk7* ^−/−^ mice exhibit lower expression of pro-inflammatory cytokines in adipose tissue and a shift in adipose tissue macrophages (ATM) toward an M2 phenotype in response HFD. **a**, **b**, **d** Expression of pro-inflammatory and anti-inflammatory cytokines in subcutaneous (**a**) and epigonadal (**b**) AT and BAT (**d**) (*n* = 6–8). Expressions of *Il*-*1β*, *Il*-*6*, *Mcp*-*1*, *Tenascin* and *Midkine* were significantly lower in both fat depots of AT*Klk7* ^−/−^ compared to control mice. *Tnf*-*α* and *Inf*-*γ* were lower in sc AT of AT*Klk7* ^−/−^ mice, but unchanged in epigonadal AT. Anti-inflammatory *Il*-*4* was increased in epi AT and in tendency in sc AT of AT*Klk7* ^−/−^ mice. AT *Il*-*10* expression was not significantly different between the genotypes. **c** Circulating levels of Mcp-1 were significantly lower after HFD in AT*Klk7* ^−/−^ mice, indicating amelioration of not only local but also systemic inflammation in the AT*Klk7* ^−/−^ mice (*n* = 7 per genotype). **e** Analysis of macrophage polarization in epigonadal AT. While total ATM percentage and M1-polarized macrophages were unaffected by HFD in AT*Klk7* ^−/−^ mice, the number of anti-inflammatory M2-polarized macrophages was significantly higher compared to control mice. This is also reflected by the significantly altered M1/M2 ratio AT*Klk7* ^−/−^ compared to control mice (*n* = 7–8 per genotype). Data are presented as mean ± SEM and for each gene expression differences between genotypes were tested for statistical significance by a two-tailed Student’s *t* test; *P < 0.05, **P < 0.01, ***P < 0.001

To analyze whether Klk7 deficiency in AT also affects HFD-induced AT inflammation, we examined the expression of marker genes of inflammation in epi and sc AT depots after HFD (Fig. 8a, b). Indeed, mRNA expression of various important pro-inflammatory cytokines such as *Tnf*-*α*, *Il*-*1β*, *Il*-*6* and *Mcp*-*1* was significantly decreased in both AT depots. Also, the expression of pro-inflammatory molecules tenascin-C (TNC) and midkine was decreased in both AT depots of AT*Klk7* ^−/−^ compared to control mice (Fig. 8a, b). Also, circulating Mcp-1 levels were significantly lower in AT*Klk7* ^−/−^ compared to control mice after HFD, reflecting systemic changes in HFD-induced inflammation (Fig. 8c). In contrast, the expression of the KLK7 substrate chemerin was unchanged in sc and epi AT and also circulating chemerin was not different in HFD-fed AT*Klk7* ^−/−^ mice (Fig. 8a, b; Table 1). JUN N-terminal kinase (JNK) family of serine/threonine protein kinases are important regulators of insulin resistance and activated by many inflammatory stimuli [27]. But JNK1 protein expression in AT was not different in HFD-fed AT*Klk7* ^−/−^ mice (Supplementary Fig. 2). mRNA expression of anti-inflammatory *Il*-*4* was significantly increased in epi AT of AT*Klk7* ^−/−^ compared to control mice, whereas no significant differences between genotypes were found for *Il*-*10* expression (Fig. 8b). In line with these findings, we also found all pro-inflammatory genes significantly downregulated in BAT of AT*Klk7* ^−/−^, while anti-inflammatory genes were similar between both genotypes (Fig. 8d).

We analyzed epigonadal ATM distribution and polarization in detail via flow cytometry. To rule out the effects of lower body weight in AT*Klk7* ^−/−^ mice, we analyzed ATMs of animals with comparable body weight (Supplementary Fig. 3). Interestingly, there was no difference in the total amount of ATM between control and AT*Klk7* ^−/−^ mice (Fig. 8e). However, while the percentage of M1 pro-inflammatory macrophages was similar in AT from both genotypes, the percentage of M2 anti-inflammatory macrophages was significantly higher in AT*Klk7* ^−/−^ mice, which therefore exhibit a less pro-inflammatory pattern of M1/M2 macrophages in epi AT (Fig. 8e). In general, the adipose tissue of HFD-fed AT*Klk7* ^−/−^ mice featured significantly reduced pro-inflammatory cytokine expression and along these lines retained a larger population of anti-inflammatory M2 macrophages, which seems to prevent a HFD-induced imbalance of M1/M2 macrophages and finally preserve whole body insulin sensitivity under HFD conditions.

## Discussion

Studies on KLK7 function in vivo have focused on inflammatory skin diseases [20, 28] and with the finding of amyloid β-peptide cleavage also Alzheimer’s disease [29]. The aim of this study was to investigate the consequences of Klk7 deficiency in AT based on the identification of the anti-diabetic and anti-inflammatory adipose tissue-derived serpin vaspin as an inhibitor of this protease [9]. We therefore generated mice with an adipose tissue-specific loss of *Klk7* to test the hypothesis that deficiency of AT KLK7 may mimic the beneficial effects of vaspin treatment on glucose metabolism and insulin sensitivity.

Previously, overexpression and knockout of AT vaspin expression in mice has been reported [11]. In accordance with vaspin transgenic (tg) mice, AT *Klk7* deficiency revealed the most striking differences between the genotypes under the condition of high fat diet-induced obesity. Both, under chow and HFD, body weight gain was reduced in AT*Klk7* ^−*/*−^ mice by ~10%. Under HFD, AT*Klk7* ^−*/*−^ mice exhibited lower epi AT weight which was accompanied by augmented storage of excess calories in sc AT, indicating altered lipid distribution into the adipose depots. Unlike in vaspin tg mice, adipocyte size in the epi AT depot was not affected by *Klk7* deficiency under both diets, but sc adipocyte size was different. While significantly smaller under chow conditions, sc adipocytes became hypertrophic after HFD in AT*Klk7* ^−*/*−^ mice. Chemerin expression in adipocytes also positively regulates adipogenesis [30], but AT expression was not affected in AT*Klk7* ^−*/*−^ mice. AT specific loss of *Fatp4* has been shown to result in adipocyte hypertrophy (both in epi and sc AT) under HFD [31]. Also, Klf4, an activator of early adipogenesis initiating transcription factor C/EBPβ, was downregulated in sc AT, further suggesting that higher expandability of sc AT in AT*Klk7* ^−*/*−^ mice is mainly due to adipocyte hypertrophy. Here, together with significantly reduced adipocyte proliferation as indicated by a ~50% decrease in *Ki*-*67* expression in all AT depots under HFD, the reduction in *Fatp4* expression in sc AT may contribute to the hypertrophy of sc adipocytes and thus prevent excessive epigonadal adipose tissue accumulation under HFD in AT*Klk7* ^−*/*−^ mice.

Analysis of in vitro differentiation and lipid accumulation of primary adipocytes from mice under chow diet revealed only minor differences between the genotypes. In line with the smaller subcutaneous adipocytes observed in vivo under chow diet, we also found significantly lower lipid accumulation in primary sc adipocytes. In contrast, while we observed increased lipid content in primary adipocytes form epi AT of AT*Klk7* ^−*/*−^ mice in vitro, the size of epigonadal adipocytes under chow diet was not different in both genotypes in vivo. In conclusion, KLK7 does not seem to significantly affect differentiation under chow conditions. In line with these findings, we have recently shown that the KLK7 inhibitor vaspin does not affect differentiation in the adipocyte model 3T3-L1, when added to the cell culture medium or when stably overexpressed [26]. In conclusion, additional and yet unknown regulatory mechanisms likely contribute to the switch in adipose tissue distribution under HFD in AT*Klk7* ^−*/*−^ mice.

Indirect calorimetry revealed increased oxygen consumption and energy expenditure for AT*Klk7* ^−*/*−^ mice under HFD. This effect was independent of activity and despite increased food intake. Furthermore, a decreased RER of 0.7 indicated improved fatty acid oxidation and better adaptation to the HFD in AT*Klk7* ^−*/*−^ mice. Lower leptin levels in AT*Klk7* ^−/−^ mice may contribute to both higher intrinsic energy expenditure and higher food intake. Also, activation of BAT and/or browning of sc AT may contribute to improved insulin sensitivity, increase in energy expenditure and body weight loss. *Ucp1* and *Pgc1α* expressions were unchanged in BAT and epi AT, both genes were increased in sc AT of AT*Klk7* ^−/−^ mice under HFD, though these increases were very inhomogeneous and not statistically significant. While our initial results presented here do not provide clear evidence that KLK7 deficiency increases BAT activity or WAT browning, the maintenance of a healthy BAT morphology (with less lipid accumulation or “whitening”) and lower BAT inflammation under HFD may indeed be beneficial for BAT activity. Future studies analyzing AT*Klk7* ^−/−^ mice after cold exposure or adrenergic stimulation will specifically address the effects of KLK7 deficiency on BAT. Also, expression of KLK7 inhibitor vaspin was increased in BAT after HFD in AT*Klk7* ^−/−^ mice, which is in line with our recent findings of BAT-specific upregulation of vaspin expression under BAT-activating stimuli of HFD and especially cold exposure [32]. Notably, while indirect calorimetry revealed a clear phenotype, it is important to note that these experiments were only performed with a small number of animals. In conclusion, increased energy expenditure is likely to underlie the leaner phenotype and may even overcompensate higher food intake in AT*Klk7* ^−*/*−^ mice.

The leptin to adiponectin ratio, which has been shown to be a very useful biomarker and predictor of the metabolic syndrome [33], is increased in both genotypes under HFD but significantly less in AT*Klk7* ^−*/*−^ mice. Accumulation of visceral AT is a risk factor for insulin resistance, type-2 diabetes and cardiovascular diseases [34–36]. Improved glucose metabolism and insulin sensitivity in AT*Klk7* ^−*/*−^ mice could therefore be mediated by an AT redistribution from epi to sc fat depots. Lower plasma insulin in AT*Klk7* ^−*/*−^ mice supports our initial hypothesis that circulating insulin is protected from KLK7-mediated degradation via inhibition by vaspin [9]. KLK7 converts prochemerin into active chemerin [37], and circulating chemerin has been shown to reduce skeletal muscle insulin sensitivity [38]. However, it is unlikely that AT is the major organ for this mechanism since AT*Klk7* ^−*/*−^ mice do not display altered chemerin serum concentrations (or AT *chemerin* expression). Increased production and secretion of pro-inflammatory cytokines such as TNF-α or IL-6 in AT dysfunction states [39] may recruit macrophages into AT and contribute to a chronic inflammatory state [40, 41]. Various mouse models deficient in (functional) genes important for ATM recruitment, such as *Tnf*-*α* or *Ccr2*, are protected from HFD-induced insulin resistance [42, 43]. Overexpression of vaspin in AT results in reduced IL-6 levels after HFD and lower expression of immune response genes in AT [11]. In AT*Klk7* ^−*/*−^ mice, we observed the most striking difference between HFD-fed AT*Klk7* ^−*/*−^ mice and littermates with respect to adipose tissue inflammation. Expression of major pro-inflammatory cytokines was significantly reduced in epi and sc AT. Importantly, amelioration of local AT inflammation in HFD-fed AT*Klk7* ^−*/*−^ mice was also reflected by changes in systemic inflammation with significantly lower Mcp1 serum levels in the AT*Klk7* ^−*/*−^ mice. Furthermore, in obesity, AT macrophages are especially increased in epi AT depots [44] and polarization of anti-inflammatory M2 macrophages, resident in AT of lean animals or humans, is switched to the pro-inflammatory M1 state under HFD and contributes to insulin resistance [45]. Analysis of ATMs in epi AT of HFD-fed AT*Klk7* ^−*/*−^ mice revealed that the number of pro-inflammatory M1 macrophages was not different. However, the number of M2 macrophages is higher in AT*Klk7* ^−*/*−^ mice, resulting in a beneficial ratio of M1/M2 ATMs reflecting a healthier AT phenotype. Along these lines, expressions of *Tnf*-*α* and of *Ifn*-*γ,* a key cytokine driving ATM polarization to change from M2 to pro-inflammatory M1 [46], were not different in epi AT of AT*Klk7* ^−*/*−^ mice. Moreover, M2 macrophage eliciting *Il*-*4* expression was significantly upregulated in epi AT of AT*Klk7* ^−*/*−^ mice, potentially contributing to the increased number of M2 macrophages remaining present in epi AT of HFD-fed AT*Klk7* ^−*/*−^ mice [47]. Vaspin expression has been shown to be increased after HFD in rat epi AT, while application or transgenic overexpression of vaspin in AT improved HFD-induced expression of pro-inflammatory genes [7, 11]. Here, the expression of *vaspin* is significantly reduced in epi AT of HFD-fed AT*Klk7* ^−*/*−^ mice. These results may further reflect attenuated inflammation in AT of AT*Klk7* ^−*/*−^ mice, as deficiency of the protease target may render increased or compensatory vaspin expression redundant.

While providing evidence for a novel and previously unrecognized role of KLK7 in adipose tissue and obesity-associated inflammation, the clear limitation of this study is that the physiological substrates of KLK7 in AT which contribute to the observed phenotype remain unknown. Yet, there is a growing number of potential peptide and protein targets that may suggest possible pathways affected by KLKs in general and KLK7 specifically (reviewed in [48]). With respect to the observed anti-inflammatory phenotype in AT of AT*Klk7* ^−*/*−^ mice, various pro-inflammatory cytokines (IL-1β [49]), adipokines (chemerin [37], midkine [50]) or other proteins (TNC [50]) or proteases (MMP9 [51]) may be relevant and have been previously shown to be substrates of KLK7. IL-1β represents an insulin resistance-promoting adipokine with strong pro-inflammatory properties and AT expression is upregulated in HFD-induced obesity and insulin-resistant mouse models [52]. IL-1β activation may be among the pro-inflammatory actions of KLK7 in AT, and AT*Klk7* ^−*/*−^ mice display a significant reduction in HFD-induced *Il*-*1β* expression in AT.

The extracellular glycoprotein TNC is a member of damage-associated molecular pattern proteins which play important roles in ECM remodeling, a key process in adipose tissue expansion and obesity [53]. Both, KLK7 and its inhibitor vaspin bind GAGs [54, 55] and may also play important roles in the process of ECM remodeling. KLK7 inhibition by vaspin is accelerated by heparin and a significant amount of secreted vaspin is localized in the ECM in human skin cells [55, 56]. KLK7 may contribute to these processes by degrading TNC as well as various other ECM proteins [48]. With this in mind, epi AT TNC expression was found to be increased in obese patients as well as in diet-induced or genetically obese mice [57] and is significantly decreased in AT of AT*Klk7* ^−*/*−^ mice after HFD.

A second limitation of this study lies within the conditional knockout of Klk7 using the Cre-recombinase under the Fabp4 promoter. Multiple studies have shown that this promoter may have unspecific effects in macrophages and also in the brain [58–60], which is important to note as the KO mice display a clear phenotype with respect to food intake, AT inflammation and ATM polarization. Although the expression of KLK7 in macrophages has not been investigated so far, using, e.g., the adiponectin promoter to achieve increased expression and AT specificity could delineate the potential effects of Klk7 deficiency in AT inflammation derived from Klk7 expressed in ATM.

**Fig. 9:**
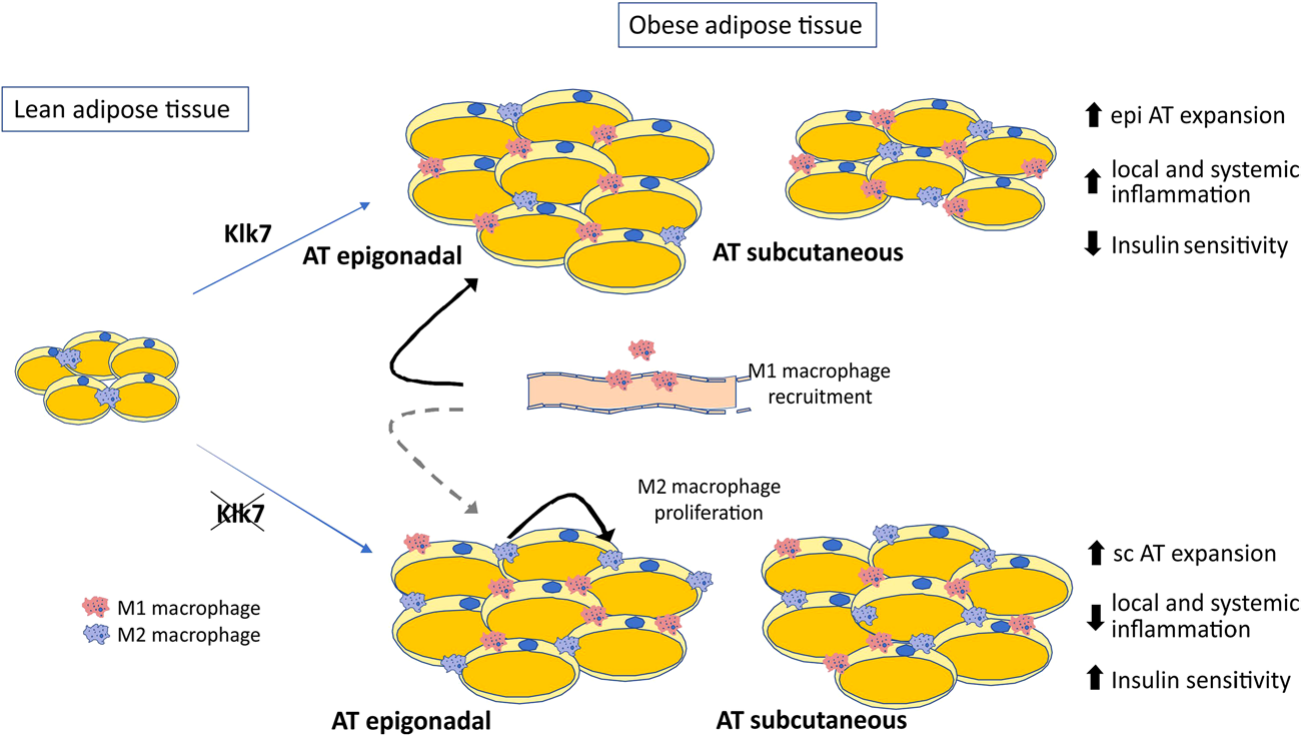
Schematic presentation of the consequences of Klk7 deficiency in adipose tissue under high fat diet conditions

In summary, our studies demonstrate that KLK7 plays an important role in AT inflammation in HFD-induced obesity and insulin resistance. The effects of AT-specific disruption of *Klk7* on insulin sensitivity may be due in part to the altered AT distribution preventing excessive epigonadal AT accumulation together with a significant reduction in AT inflammation (Fig. 9). The mechanisms underlying the effects of *Klk7* deficiency on energy expenditure remain not well understood, but could be caused by alterations in adipokine patterns in AT*Klk7* ^−*/*−^ mice. In addition, our data suggest a previously unrecognized role of KLK7 in AT inflammation. We propose KLK7 as a potential target and suggest (small compound) KLK7 inhibitors as promising therapeutic tools to improve obesity-related metabolic disorders.

## Electronic supplementary material

Below is the link to the electronic supplementary material.

Supplementary material 1 (DOCX 497 kb)

Supplementary material 2 (PDF 184 kb)

## Abbreviations

KLK7: Kallikrein 7
AT: Adipose tissue
HFD: high fat diet
ATM: Adipose tissue macrophage

## References

[1] Bluher M (2009). Adipose tissue dysfunction in obesity. Exp Clin Endocr Diab.

[2] Kahn SE, Hull RL, Utzschneider KM (2006). Mechanisms linking obesity to insulin resistance and type 2 diabetes. Nature.

[3] D’Agati VD, Chagnac A, Vries APJd, Levi M, Porrini E, Herman-Edelstein M, Praga M (2016). Obesity-related glomerulopathy: clinical and pathologic characteristics and pathogenesis. Nat Rev Nephrol.

[4] Galic S, Oakhill JS, Steinberg GR (2010). Adipose tissue as an endocrine organ. Mol Cell Endocrinol.

[5] Bluher M (2012). Vaspin in obesity and diabetes: pathophysiological and clinical significance. Endocrine.

[6] Heiker JT (2014). Vaspin (serpinA12) in obesity, insulin resistance, and inflammation. J Pept Sci.

[7] Hida K, Wada J, Eguchi J, Zhang H, Baba M, Seida A, Hashimoto I, Okada T, Yasuhara A, Nakatsuka A, Shikata K, Hourai S, Futami J, Watanabe E, Matsuki Y, Hiramatsu R, Akagi S, Makino H, Kanwar YS (2005). Visceral adipose tissue-derived serine protease inhibitor: a unique insulin-sensitizing adipocytokine in obesity. PNAS.

[8] Kloting N, Kovacs P, Kern M, Heiker JT, Fasshauer M, Schon MR, Stumvoll M, Beck-Sickinger AG, Bluher M (2011). Central vaspin administration acutely reduces food intake and has sustained blood glucose-lowering effects. Diabetologia.

[9] Heiker JT, Kloting N, Kovacs P, Kuettner EB, Strater N, Schultz S, Kern M, Stumvoll M, Bluher M, Beck-Sickinger AG (2013). Vaspin inhibits kallikrein 7 by serpin mechanism. Cell Mol Life Sci.

[10] Hida K, Wada J, Zhang H, Hiragushi K, Tsuchiyama Y, Shikata K, Makino H (2000). Identification of genes specifically expressed in the accumulated visceral adipose tissue of OLETF rats. J Lipid Res.

[11] Nakatsuka A, Wada J, Iseda I, Teshigawara S, Higashio K, Murakami K, Kanzaki M, Inoue K, Terami T, Katayama A, Hida K, Eguchi J, Horiguchi CS, Ogawa D, Matsuki Y, Hiramatsu R, Yagita H, Kakuta S, Iwakura Y, Makino H (2012). Vaspin is an adipokine ameliorating ER stress in obesity as a ligand for cell-surface GRP78/MTJ-1 complex. Diabetes.

[12] Yousef GM, Chang A, Scorilas A, Diamandis EP (2000). Genomic organization of the human kallikrein gene family on chromosome 19q13.3-q13.4. Biochem Biophys Res Com.

[13] Clements JA (2008). Reflections on the tissue kallikrein and kallikrein-related peptidase family—from mice to men—what have we learnt in the last two decades?. Biol Chem.

[14] Emami N, Diamandis EP (2007). New insights into the functional mechanisms and clinical applications of the kallikrein-related peptidase family. Mol Oncol.

[15] Goettig P, Magdolen V, Brandstetter H (2010). Natural and synthetic inhibitors of kallikrein-related peptidases (KLKs). Biochimie.

[16] Prassas I, Eissa A, Poda G, Diamandis EP (2015). Unleashing the therapeutic potential of human kallikrein-related serine proteases. Nat Rev Drug Discov.

[17] Egelrud T, Lundstrom A (1991). A chymotrypsin-like proteinase that may be involved in desquamation in plantar stratum corneum. Arch Dermatol Res.

[18] Lundstrom A, Egelrud T (1991). Stratum corneum chymotryptic enzyme: a proteinase which may be generally present in the stratum corneum and with a possible involvement in desquamation. Acta Derm Venereol.

[19] Hansson L, Backman A, Ny A, Edlund M, Ekholm E, Ekstrand Hammarstrom B, Tornell J, Wallbrandt P, Wennbo H, Egelrud T (2002). Epidermal overexpression of stratum corneum chymotryptic enzyme in mice: a model for chronic itchy dermatitis. J Invest Dermatol.

[20] Ny A, Egelrud T (2004). Epidermal hyperproliferation and decreased skin barrier function in mice overexpressing stratum corneum chymotryptic enzyme. Acta Derm Venereol.

[21] Ekholm E, Egelrud T (1999). Stratum corneum chymotryptic enzyme in psoriasis. Arch Dermatol Res.

[22] Yamasaki K, Di Nardo A, Bardan A, Murakami M, Ohtake T, Coda A, Dorschner RA, Bonnart C, Descargues P, Hovnanian A, Morhenn VB, Gallo RL (2007). Increased serine protease activity and cathelicidin promotes skin inflammation in rosacea. Nat Med.

[23] Kern M, Kosacka J, Hesselbarth N, Bruckner J, Heiker JT, Flehmig G, Kloting I, Kovacs P, Matz-Soja M, Gebhardt R, Krohn K, Sales S, Abshagen K, Shevchenko A, Stumvoll M, Bluher M, Kloting N (2014). Liver-restricted repin1 deficiency improves whole-body insulin sensitivity, alters lipid metabolism, and causes secondary changes in adipose tissue in mice. Diabetes.

[24] Haase J, Weyer U, Immig K, Kloting N, Bluher M, Eilers J, Bechmann I, Gericke M (2014). Local proliferation of macrophages in adipose tissue during obesity-induced inflammation. Diabetologia.

[25] Petrovic N, Shabalina IG, Timmons JA, Cannon B, Nedergaard J (2008). Thermogenically competent nonadrenergic recruitment in brown preadipocytes by a PPARgamma agonist. Am J Physiol Endocrinol Metab.

[26] Zieger K, Weiner J, Krause K, Schwarz M, Kohn M, Stumvoll M, Bluher M, Heiker JT (2017). Vaspin suppresses cytokine-induced inflammation in 3T3-L1 adipocytes via inhibition of NFkappaB pathway. Mol Cell Endocrinol.

[27] Hirosumi J, Tuncman G, Chang L, Gorgun CZ, Uysal KT, Maeda K, Karin M, Hotamisligil GS (2002). A central role for JNK in obesity and insulin resistance. Nature.

[28] Ny A, Egelrud T (2003). Transgenic mice over-expressing a serine protease in the skin: evidence of interferon gamma-independent MHC II expression by epidermal keratinocytes. Acta Derm Venereol.

[29] Shropshire TD, Reifert J, Rajagopalan S, Baker D, Feinstein SC, Daugherty PS (2014). Amyloid beta peptide cleavage by kallikrein 7 attenuates fibril growth and rescues neurons from Abeta-mediated toxicity in vitro. Biol Chem.

[30] Goralski KB, McCarthy TC, Hanniman EA, Zabel BA, Butcher EC, Parlee SD, Muruganandan S, Sinal CJ (2007). Chemerin, a novel adipokine that regulates adipogenesis and adipocyte metabolism. J Biol Chem.

[31] Lenz LS, Marx J, Chamulitrat W, Kaiser I, Grone HJ, Liebisch G, Schmitz G, Elsing C, Straub BK, Fullekrug J, Stremmel W, Herrmann T (2011). Adipocyte-specific inactivation of Acyl-CoA synthetase fatty acid transport protein 4 (Fatp4) in mice causes adipose hypertrophy and alterations in metabolism of complex lipids under high fat diet. J Biol Chem.

[32] Weiner J, Rohde K, Krause K, Zieger K, Kloting N, Kralisch S, Kovacs P, Stumvoll M, Bluher M, Bottcher Y, Heiker JT (2017). Brown adipose tissue (BAT) specific vaspin expression is increased after obesogenic diets and cold exposure and linked to acute changes in DNA-methylation. Mol Metab.

[33] Falahi E, Khalkhali Rad AH, Roosta S (2015). What is the best biomarker for metabolic syndrome diagnosis?. Diabetes Metab Syndr.

[34] Ohlson LO, Larsson B, Svardsudd K, Welin L, Eriksson H, Wilhelmsen L, Bjorntorp P, Tibblin G (1985). The influence of body fat distribution on the incidence of diabetes mellitus. 13.5 years of follow-up of the participants in the study of men born in 1913. Diabetes.

[35] Mori Y, Hoshino K, Yokota K, Yokose T, Tajima N (2005). Increased visceral fat and impaired glucose tolerance predict the increased risk of metabolic syndrome in Japanese middle-aged men. Exp Clin Endocr Diab.

[36] Basat O, Ucak S, Ozkurt H, Basak M, Seber S, Altuntas Y (2006). Visceral adipose tissue as an indicator of insulin resistance in nonobese patients with new onset type 2 diabetes mellitus. Exp Clin Endocr Diab.

[37] Schultz S, Saalbach A, Heiker JT, Meier R, Zellmann T, Simon JC, Beck-Sickinger AG (2013). Proteolytic activation of prochemerin by kallikrein 7 breaks an ionic linkage and results in C-terminal rearrangement. Biochem J.

[38] Sell H, Laurencikiene J, Taube A, Eckardt K, Cramer A, Horrighs A, Arner P, Eckel J (2009). Chemerin is a novel adipocyte-derived factor inducing insulin resistance in primary human skeletal muscle cells. Diabetes.

[39] Hotamisligil GS, Shargill NS, Spiegelman BM (1993). Adipose expression of tumor necrosis factor-alpha: direct role in obesity-linked insulin resistance. Science.

[40] Xu H, Barnes GT, Yang Q, Tan G, Yang D, Chou CJ, Sole J, Nichols A, Ross JS, Tartaglia LA, Chen H (2003). Chronic inflammation in fat plays a crucial role in the development of obesity-related insulin resistance. J Clin Invest.

[41] Weisberg SP, McCann D, Desai M, Rosenbaum M, Leibel RL, Ferrante AW (2003). Obesity is associated with macrophage accumulation in adipose tissue. J Clin Invest.

[42] Uysal KT, Wiesbrock SM, Marino MW, Hotamisligil GS (1997). Protection from obesity-induced insulin resistance in mice lacking TNF-alpha function. Nature.

[43] Weisberg SP, Hunter D, Huber R, Lemieux J, Slaymaker S, Vaddi K, Charo I, Leibel RL, Ferrante AW (2006). CCR2 modulates inflammatory and metabolic effects of high-fat feeding. J Clin Invest.

[44] Harman-Boehm I, Bluher M, Redel H, Sion-Vardy N, Ovadia S, Avinoach E, Shai I, Kloting N, Stumvoll M, Bashan N, Rudich A (2007). Macrophage infiltration into omental versus subcutaneous fat across different populations: effect of regional adiposity and the comorbidities of obesity. J Clin Endocrinol Metab.

[45] Lumeng CN, Bodzin JL, Saltiel AR (2007). Obesity induces a phenotypic switch in adipose tissue macrophage polarization. J Clin Invest.

[46] Goldszmid RS, Caspar P, Rivollier A, White S, Dzutsev A, Hieny S, Kelsall B, Trinchieri G, Sher A (2012). NK cell-derived interferon-gamma orchestrates cellular dynamics and the differentiation of monocytes into dendritic cells at the site of infection. Immunity.

[47] Liao XD, Sharma N, Kapadia F, Zhou GJ, Lu Y, Hong H, Paruchuri K, Mahabeleshwar GH, Dalmas E, Venteclef N, Flask CA, Kim J, Doreian BW, Lu KQ, Kaestner KH, Hamik A, Clement K, Jain MK (2011). Kruppel-like factor 4 regulates macrophage polarization. J Clin Invest.

[48] Yu YJ, Prassas I, Diamandis EP (2014). Putative kallikrein substrates and their (patho) biological functions. Biol Chem.

[49] NylanderLundqvist E, Egelrud T (1997). Formation of active IL-1 beta from pro-IL-1 beta catalyzed by stratum corneum chymotryptic enzyme in vitro. Acta Derm Venereol.

[50] Yu Y, Prassas I, Dimitromanolakis A, Diamandis EP (2015). Novel biological substrates of human kallikrein 7 identified through degradomics. J Biol Chem.

[51] Ramani VC, Kaushal GP, Haun RS (2011). Proteolytic action of kallikrein-related peptidase 7 produces unique active matrix metalloproteinase-9 lacking the C-terminal hemopexin domains. Biochim Biophys Acta.

[52] Lagathu C, Yvan-Charvet L, Bastard JP, Maachi M, Quignard-Boulange A, Capeau J, Caron M (2006). Long-term treatment with interleukin-1 beta induces insulin resistance in murine and human adipocytes. Diabetologia.

[53] Mariman ECM, Wang P (2010). Adipocyte extracellular matrix composition, dynamics and role in obesity. Cell Mol Life Sci.

[54] Oliveira JR, Bertolin TC, Andrade D, Oliveira LC, Kondo MY, Santos JA, Blaber M, Juliano L, Severino B, Caliendo G, Santagada V, Juliano MA (2015). Specificity studies on Kallikrein-related peptidase 7 (KLK7) and effects of osmolytes and glycosaminoglycans on its peptidase activity. Biochem Biophys Acta.

[55] Ulbricht D, Oertwig K, Arnsburg K, Saalbach A, Pippel J, Strater N, Heiker JT (2017). Basic residues of beta-sheet a contribute to heparin binding and activation of vaspin (Serpin A12). J Biol Chem.

[56] Saalbach A, Tremel J, Herbert D, Schwede K, Wandel E, Schirmer C, Anderegg U, Beck-Sickinger AG, Heiker JT, Schultz S, Magin T, Simon JC (2016). Anti-inflammatory action of keratinocyte-derived vaspin: relevance for the pathogenesis of psoriasis. Am J Pathol.

[57] Catalan V, Gomez-Ambrosi J, Rodriguez A, Ramirez B, Rotellar F, Valenti V, Silva C, Gil MJ, Salvador J, Fruhbeck G (2012). Increased tenascin C and Toll-like receptor 4 levels in visceral adipose tissue as a link between inflammation and extracellular matrix remodeling in obesity. J Clin Endocrinol Metab.

[58] Lee KY, Russell SJ, Ussar S, Boucher J, Vernochet C, Mori MA, Smyth G, Rourk M, Cederquist C, Rosen ED, Kahn BB, Kahn CR (2013). Lessons on conditional gene targeting in mouse adipose tissue. Diabetes.

[59] Jeffery E, Berry R, Church CD, Yu S, Shook BA, Horsley V, Rosen ED, Rodeheffer MS (2014). Characterization of Cre recombinase models for the study of adipose tissue. Adipocyte.

[60] Mullican SE, Tomaru T, Gaddis CA, Peed LC, Sundaram A, Lazar MA (2013). A novel adipose-specific gene deletion model demonstrates potential pitfalls of existing methods. Mol Endocrinol.

