# Supplementary material for: Ablation of kallikrein 7 (KLK7) in adipose tissue ameliorates metabolic consequences of high fat diet-induced obesity by counteracting adipose tissue inflammation in vivo
Source: Cell Mol Life Sci. 2017 Sep 20;75(4):727–42. doi: 10.1007/s00018-017-2658-y (PMC5769829; doi:10.1007/s00018-017-2658-y)
Supplement: Supplementary file 1 — Supplementary material 1 (DOCX 497 kb) [file 18_2017_2658_MOESM1_ESM.docx]

**SUPPLEMENTAL MATERIAL**

**Ablation of kallikrein 7 (KLK7) in adipose tissue ameliorates metabolic consequences of high-fat diet induced obesity by counteracting adipose tissue inflammation *in vivo***

Konstanze Zieger, Juliane Weiner, Anne Kunath, Martin Gericke, Kerstin Krause, Matthias Kern, Michael Stumvoll, Nora Klöting, Matthias Blüher, and John T. Heiker

**Supplementary Table 1**. Primer sequences used for RNA expression analysis

| **Gene** | **Forward** | **Reverse** |
| --- | --- | --- |

*36b4* AAGCGCGTCCTGGCATTGTCT CCGCAGGGGCAGCAGTGGT

*Pparγ* CGTGAAGCCCATCGAGGACATC TGGAGCAGGGGGTGAAG

*Ki-67* TGTGAGGCTGAGACATGGAG CCTTGATGGTTCCTTTCCAA

*Klf4* GACTAACCGTTGGCGTGAGG GTCTAGGTCCAGGAGGTCGT

*Fatp4* ATTGTGGTGCACAGCAGGTAT AGTCATGCCGTGGAGTAAGC

*Il-1β* TGCCACCTTTTGACAGTGATG AAGGTCCACGGGAAAGACAC

*Il-6* ACCTGGAGTACATGAAGAACA TTGGAAATTGGGGTAGGAAG

*Il-4* CCATATCCACGGATGCGACA CTGTGGTGTTCTTCGTTGCTG

*Il-10* GGCGCTGTCATCGATTTCTC CGGAGAGAGGTACAAACGAGG

*Mcp-1* GCCCCACTCACCTGCTGCTACT CCTGCTGCTGGTGATCCTCTTGT

*Midkine* TGGAGCCGACTGCAAATACAA GGCTTAGTCACGCGGATGG

*Inf-γ* AGGAACTGGCAAAAGGATGGT TCATTGAATGCTTGGCGCTG

*Tnf-α* GTCCCCAAAGGGATGAGAAGT GCTCCTCCACTTGGTGGTTT

*Tnc-c* CAGCTACCGACGGGATCTTC TTCCGGTTCAGCTTCTGTGG

*Chemerin* TACAGGTGGCTCTGGAGGAGTTC CTTCTCCCGTTTGGTTTGATTG

*Pgc1α*  CTTTTGTGGACGGAAGCAAT GAGTCTTGGGAAAGGACACG

*Vaspin* CCGCATCTCGTCTACTTAC CTCCCACCTTCAGGCTTC

*Ucp1*  CCGAAACTGTACAGCGGTCT CCGAGAGAGGCAGGTGTTTC

*Pref1* TGT GCA GGA GCA TTC GTA CT CGG GAA ATT CTG CGA AAT AG

**Supplementary Figure 1**


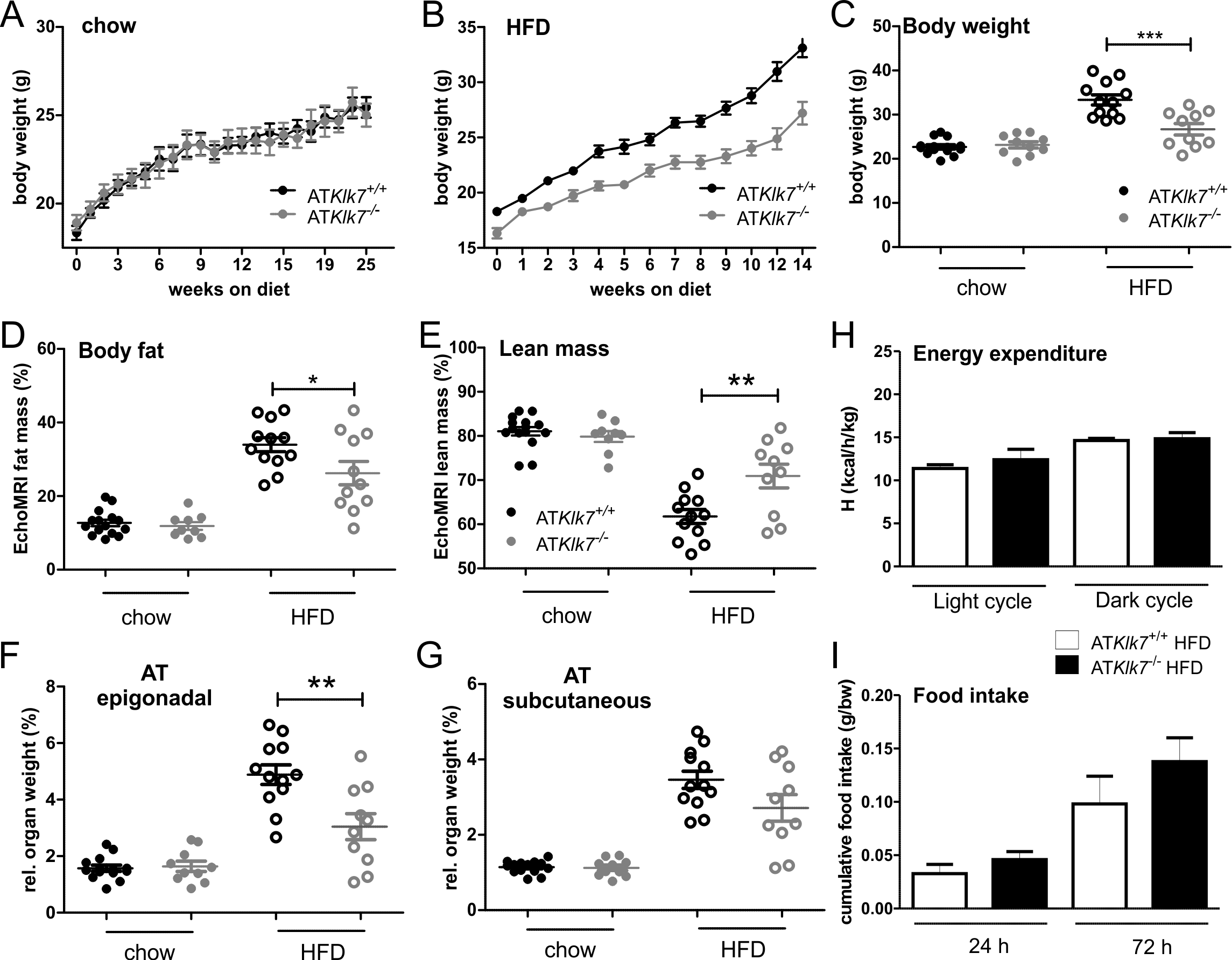


**Supplementary Figure 1. Klk7-deficiency in adipose tissue in female mice under chow and high fat diet.** (A, B) Body weight gain and (C) final body weight of ATKlk7^-/-^ and control littermates fed chow diet or HFD (B), starting at the age of 6 weeks (n=9-16). (D, E) The percentage of fat mass and lean body mass as determined by EchoMRI at the end of the studies under chow (at age 26 weeks) and HFD conditions (20 weeks of age; n=10-12). (F-H) Relative organ weights of epigonadal and subcutaneous WAT depots (n=8-14) at the end of chow and HFD studies. ATKlk7^-/-^ exhibit altered fat distribution with decreased epigonadal AT mass. (H-I) Net energy expenditure (H) and cumulative food intake (I) is slightly higher in ATKlk7^-/-^ compared to controls. Data are represented as mean ± SEM and for each diet condition, differences between genotypes were tested for statistical significance by a two-tailed Student´s t-test; *P ˂ 0.05, ** P ˂ 0.01, *** P ˂ 0.001.

**Supplementary Figure 2**


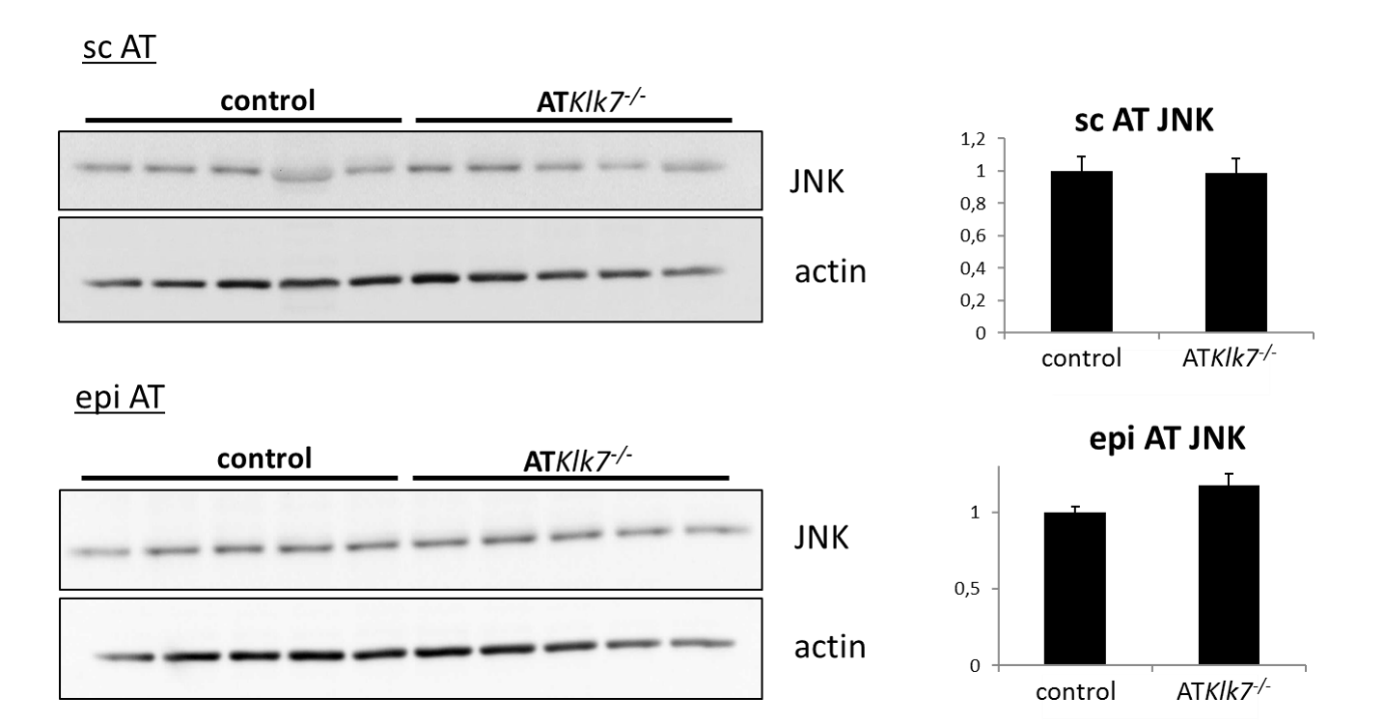


**Supplementary Figure 2. JNK expression in sc (top) and epi (bottom) AT of control and AT*KLK7^-/-^* mice after HFD**. Western Blot analyses (left) and quantification of band intensities (right) in AT of n=5 animals. JNK band intensities were normalized against actin band.

**Supplementary Figure 3**


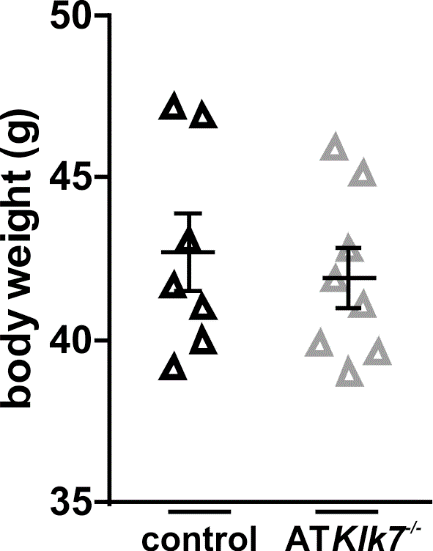


**Supplementary Figure 3. Body weights of HFD-fed control and AT*KLK7^-/-^* mice used for adipose tissue macrophage characterization**. n=6 animals with matched weights were used in these experiments to preclude body weight effects.
