# Supplementary material for: Ablation of kallikrein 7 (KLK7) in adipose tissue ameliorates metabolic consequences of high fat diet-induced obesity by counteracting adipose tissue inflammation in vivo
Source: Cell Mol Life Sci. 2017 Sep 20;75(4):727–42. doi: 10.1007/s00018-017-2658-y (PMC5769829; doi:10.1007/s00018-017-2658-y)
Supplement: Supplementary file 2 — Supplementary material 2 (PDF 184 kb) [file 18_2017_2658_MOESM2_ESM.pdf]

Figure	N WT chow	N KO chow	N WT HFD	N KO HFD
2 A	14	10	-	-
2 B	-	-	16	12
2 C	14	10	12	12
2 D	13	9	12	12
2 E	13	10	16	12
2 F	14	8	12	12
2 G	14	8	12	12
2 H	14	8	12	12
3	-	-	4	4
4 A	13	10	11	10
4 B	12	8	10	12
4 C	5	9	n.d.	n.d.
4 D	8	10	9	8
4 E	10	9	10	9
4 F	8	9	9	8
5 B epiAT	14	10	12	12
5 B scAT	14	9	10	9
8 C	-	-	7	7
8 E total ATM	-	-	7	8
8 E M1	-	-	7	8
8 E M2	-	-	7	8
8 E M1/M2	-	-	7	8

Table 1	N WT chow	N KO chow	N WT HFD	N KO HFD
adipoq	8	10	10	10
leptin	10	10	10	10
HbA1c	13	10	16	12
chemerin	-	-	8	8
FFA	10	10	10	10
totalcholester	10	10	10	10
triglycerides	10	10	10	10
glucose	13	9	12	12

	WT		KO	
	primary epi	primary sc	primary epi	primary sc
6 A, B	5	5	5	5
6 C, D	3 / 3	3 / 3	3 / 3	3 / 3

		scAT		epiAT		BAT	
	AT genes	N WT HFD	N KO HFD	N WT HFD	N KO HFD	N WT HFD	N KO HFD
Figure 8 A, B, D	TNFa	7	7	8	8	8	8
	IL6	6	7	8	7	8	7
	MCP1	6	8	7	8	8	7
	IL1B	7	8	8	8	8	8
	Tenascin	7	8	8	8	8	8
	INFg	6	8	7	8	8	8
	Midkine	4	2	5	6	7	4
	IL10	6	8	8	8	8	8
	IL4	6	7	7	7	8	8
	Chemerin	7	7	7	6	8	8
	vaspin	7	6	8	7	8	8
5 C / 7 B	Pparg	7	8	8	8	8	8
5 D / 7 B	Ki67	6	8	8	7	8	7
5 E / 7 B	Klf4	7	8	7	8	8	7
5 F / 7 B	Fatp4	7	8	8	8	8	8
5 G / 7 B	Vaspin	6	7	8	7	8	8
7 C	UCP1	7	6	8	7	8	8
7 D	PGC1a	5	3	7	6	8	8
